# Collective quantum coherence and subband redistribution in artificially assembled nanotube arrays

**DOI:** 10.1093/nsr/nwag052

**Published:** 2026-01-27

**Authors:** Xiao-Song Deng, Wei-Li Li, Hao-Yu Zhang, Xiao-Han Cheng, Zi-Xuan Zhang, Guan-Hua Long, Chen-Wei Fan, Zheng-Shan Guo, Tian Pei, Chuan-Hong Jin, Sheng Wang, Yan-Ning Zhang, Ning Kang, Zhi-Yong Zhang

**Affiliations:** Key Laboratory for the Physics and Chemistry of Nanodevices and Center for Carbon-based Electronics, School of Electronics, Peking University, Beijing 100871, China; Institute of Fundamental and Frontier Sciences, Key Laboratory of Quantum Physics and Photonic Quantum Information of Ministry of Education, University of Electronic Science and Technology of China, Chengdu 610054, China; Key Laboratory for the Physics and Chemistry of Nanodevices and Center for Carbon-based Electronics, School of Electronics, Peking University, Beijing 100871, China; Key Laboratory for the Physics and Chemistry of Nanodevices and Center for Carbon-based Electronics, School of Electronics, Peking University, Beijing 100871, China; State Key Laboratory of Silicon Materials and Advanced Semiconductor Materials, School of Materials Science and Engineering, Zhejiang University, Hangzhou 310027, China; Key Laboratory for the Physics and Chemistry of Nanodevices and Center for Carbon-based Electronics, School of Electronics, Peking University, Beijing 100871, China; Key Laboratory for the Physics and Chemistry of Nanodevices and Center for Carbon-based Electronics, School of Electronics, Peking University, Beijing 100871, China; Beijing Academy of Quantum Information Science, Beijing 100193, China; Beijing Academy of Quantum Information Science, Beijing 100193, China; State Key Laboratory of Silicon Materials and Advanced Semiconductor Materials, School of Materials Science and Engineering, Zhejiang University, Hangzhou 310027, China; Key Laboratory for the Physics and Chemistry of Nanodevices and Center for Carbon-based Electronics, School of Electronics, Peking University, Beijing 100871, China; Institute of Fundamental and Frontier Sciences, Key Laboratory of Quantum Physics and Photonic Quantum Information of Ministry of Education, University of Electronic Science and Technology of China, Chengdu 610054, China; Key Laboratory for the Physics and Chemistry of Nanodevices and Center for Carbon-based Electronics, School of Electronics, Peking University, Beijing 100871, China; Hefei National Laboratory, Hefei 230088, China; Key Laboratory for the Physics and Chemistry of Nanodevices and Center for Carbon-based Electronics, School of Electronics, Peking University, Beijing 100871, China

**Keywords:** quantum transport, electronic coherence, carbon nanotube, intertube coupling

## Abstract

Artificial assembly of one-dimensional ballistic conductors into a two-dimensional (2D) system can provide an ideal platform to study coherent electronic coupling and designable physical properties. However, systematic investigations of both the coupling and ballistics in such artificially assembled systems remain scarce. Here, we report collective quantum coherence in a quasi-2D film consisting of well-aligned single-walled carbon nanotubes (CNTs) with intertube coupling. The conductance plateaus in the quasi-ballistic regime demonstrate subband occupations of hundreds of CNTs in a collective manner. The experimental observations agree with density functional theory simulations considering subband redistribution with intertube coupling. Finally, we summarize the quantum coherent transport for multichannel coupled systems in distinct regimes. These results open an avenue towards exploring engineered artificial systems for coherent electronic devices and hold potential for the development of novel high-performance and quantum nanoelectronics.

## Teaser

Intertube coupling in artificially assembled carbon nanotube arrays enables collective quantum behaviors, advancing engineered quantum nanoelectronic devices.

## INTRODUCTION

Understanding and engineering electronic systems with periodic structural modulation are of great fundamental and practical interest. Typically, analogous to the formation of crystals, the periodic arrangement of atoms and the collective motion of electrons define the band structure of crystals and thus their physical properties. Therefore, studying the role of coherent coupling in artificial systems is important for customizing their electronic properties and designing quantum simulators. Advances in material growth and fabrication strategies have led to systems with promising artificially controlled coupling and material properties, such as semiconductor superlattices [1,2], artificial one-dimensional (1D) superlattices in two-dimensional electron gas (2DEG) systems [3,4], quasi-one-dimensional (quasi-1D) nanowires with a finite diameter or nanowire bundles [5–8], and interlayer-coupled two-dimensional (2D) moiré patterns [9–11], in which rich quantum transport phenomena and correlated phase diagrams arise. Nevertheless, systematic studies of the coupling effects and electronic properties in coupled 1D systems remain scarce owing to the challenging construction of uniform arrays with large areas and high-quality devices.

**Figure 1.**
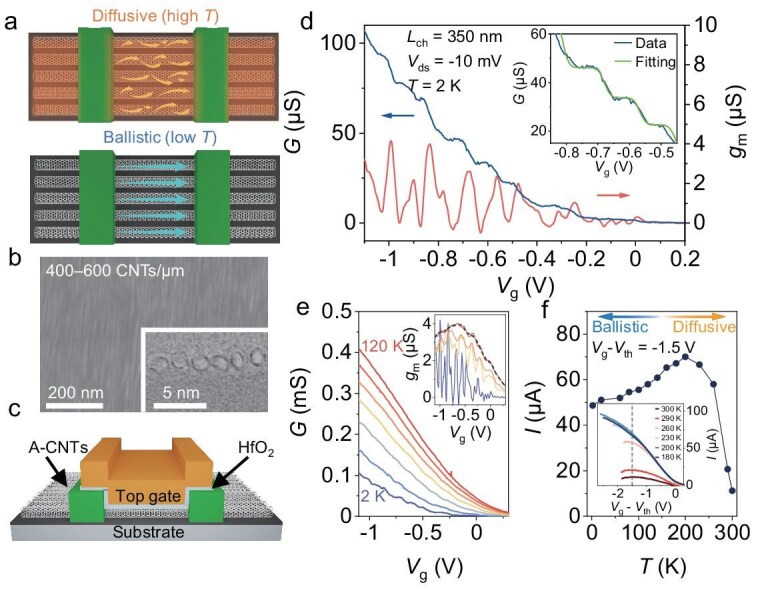
Conductance quantization in an A-CNT device. (a) Schematic illustrations of diffusive transport at high temperatures and ballistic transport with suppressed electron‒phonon scattering at low temperatures. (b) SEM image and TEM image of A-CNT films with a density of 400–600 CNTs/μm. (c) Structure of a top-gate FET based on A-CNT films. (d) Transfer curve and transconductance curve at 2 K, indicating multiple conductance quantization. Inset: experimental fitting via the generalized Landauer–Büttiker formalism with the *c* of 1.7 meV. (e) Transfer curves at various temperatures ranging from 2 to 120 K at *V*_ds_ = −10 mV. Inset: transconductance curves obtained from the main plot. (f) Current as a function of temperature from 300 to 2 K at *V*_ds_ = −100 mV and *V*_g_−*V*_th_ = −1.5 V. Inset: transfer curves at various temperatures, normalized by *V*_th_. The dashed line indicates the position of *V*_g_−*V*_th_ = −1.5 V to extract the current for the main plot. The current drops at on-state regime and high temperature originate from the trapped charges in the gate stack. The data in (a–f) are obtained from the same device.

The observation of pronounced coupling effects requires reduction of the disorder and suppression of carrier scattering in channels. Decreasing the channel length or temperature is a typical strategy for converting solid-state systems from diffusive to ballistic transport [12], as shown in Fig. 1a. With the mitigation of thermal fluctuations at low temperatures, the ballistic transport in low-dimensional systems can be verified by observations of characteristic transport phenomena sensitive to destructive scattering, such as subband occupation [13], electronic coherence [14], and correlated states [15], in addition to the demonstration of conductivity limits with perfect transmission [16].

Carbon nanotubes (CNTs), as ideal ballistic conductors with excellent electronic properties, are a promising platform for prototype devices [17,18] and studies of low-dimensional transport [19]. The effects of coupling between CNTs can be classified into two types: coherent tunnel coupling and electrodynamic coupling. Unless otherwise stated, intertube coupling here refers to coherent tunnelling. The former coupling leads to the modification of band structure, including the formation of a pseudogap [5,6], lifting of band degeneracy [20], and formation of additional dispersion [21]; the latter type affecting the Coulomb interaction within CNTs [22–24]. However, the realization of controlled intertube coupling and phase transitions in coupled 1D arrays [25] is challenging because of the geometrical configuration of CNT bundles. To meet the requirements of large-scale integrated circuits and a sufficient driving ability, aligned-CNT (A-CNT) films with a high semiconducting purity have been constructed and present promising prospects in electronics [26]; however, the knowledge of the physical and electronic properties of such artificially assembled materials is in its infancy. A-CNT films expected to operate in the ballistic regime favor the systematic understanding of intertube coupling. The characteristics of A-CNT films, including robust 1D parallel channels, equivalent contacts and field efficiency of the most coupled channels, number of coupled channels, and efficient gate stacks, promote correlated physics and electronic applications with high performance and scalable fabrication [27–29].

In this article, we report quasi-ballistic transport and quantum coherence phenomena in 2D A-CNT films. The observed conductance plateaus indicate synchronous occupations of the subbands in the quasi-ballistic regime. We discuss the redistribution of subbands and spatial coherence of A-CNT films compared with those of isolated CNTs by performing a quantitative analysis based on nonlinear transport spectroscopy. We construct a simulation model using density functional theory (DFT) to capture the experimental transport characteristics and verify the band redistribution of A-CNT films. Ballistic transport and quantum coherence realization are further demonstrated by the observation of Fano interference in A-CNT films. Finally, we discuss the distinct behaviors of the quantum coherence phenomena in different regimes depending on the coupling strength of A-CNT films.

## RESULTS

### Conductance plateaus in the quasi-ballistic regime

Semiconducting CNT films with a parallel arrangement were assembled via the dimension-limited self-alignment method (DLSA; for details, see Supplementary Texts 1) [26]. The density of A-CNT films can be modulated in the range of 200–600 CNTs/μm by applying an appropriate lifting speed. Figure 1b shows scanning electron microscopy (SEM) and transmission electron microscopy (TEM) images of the high-density A-CNT films (400–600 CNTs/μm) with an average tube length of 1.23 ± 0.55 μm; statics results of the SEM image are shown in Fig. S1. Monolayer CNTs with a diameter of ∼1.51 ± 0.09 nm (obtained from the optical measurements in Figs S2 and S3) and an intertube spacing of < 0.5 nm (edge-to-edge) are uniformly distributed on the substrate. The A-CNT films were carefully pre-processed to decrease disorders before device construction (for details, see Methods). The structure of the device is schematically shown in Fig. 1c. The device was fabricated via a standard top-gate field-effect transistor (FET) process with thin high-κ HfO_2_ as a gate oxide layer and a doping-free method as shown in Figs S4 and S5 (for details, see Methods) [30]. The uniformity of the device behavior and the transport ability proportional to the channel widths demonstrate that a large amount of individual CNTs contribute to the transport in FETs based on the A-CNT films, as shown in Figs S6–S8. All devices in this work have the same channel width of 800 nm unless otherwise stated, to attenuate the effects of alignment fluctuations [31].

The transfer curve of a device with a channel length of *L*_ch_ = 350 nm measured at 2 K as a function of the gate voltage *V*_g_ is shown in Fig. 1d (blue line). The conductance *G* significantly increases as *V*_g_ decreases to below −0.2 V, whereas it is almost pinched off at *V*_g_ > 0 V. The drastic modulation of *G* indicates a *V*_g_-controlled shift of the Fermi level from the bandgap to the valence band. In the on-state regime, clear multiple step-like conductance plateaus appear on the transfer curve below −0.2 V. To more clearly identify the plateaus, the transconductance *g*_m_, obtained by taking the derivative of the current *I* with respect to *V*_g_, is shown in Fig. 1d (red line). The *V*_g_ intervals between conductance plateaus, ∆*V*_g_, can be extracted from the minima in *g*_m_ and ranges from 50 to 110 mV (Fig. S9). Moreover, the conductance plateaus are gradually smoothed out with increasing temperature, as shown in Fig. 1e, whereas their positions, identified based on the *g*_m_ curves in the inset of Fig. 1e, are not significantly shifted with respect to *V*_g_. The plateaus become almost indiscernible at ∼100 K. The observed phenomena are robust and cannot be attributed to some randomly formed quantum dots (QDs) or tunnelling events.

Similar conductance plateaus have been reported in some 1D and quasi-1D systems, such as individual CNTs [13,32], nanowires [7,8], and nanoribbons [33,34], where a subband configuration is formed due to the transverse quantum confinement of the system geometry. In 2DEG systems, subband construction can also be realized by an electrostatically defined quantum point contact (QPC) [35–37]. As the Fermi level crosses the subband edge, the transport channels increase, and the quantized conductance plateaus are observed as integer values of *G*_0_ × *T*_i_ = 2*e*^2^/*h* × *T*_i_, where *e* is the electron charge, *h* is the Planck constant, *T*_i_ is the transmission of the *i*th channel, and 2 is the spin degeneracy. Such experimental observations of subband occupation require the systems to operate in the ballistic regime in the channel. Ballistic transport involving multiple channels can be described by the generalized Landauer–Büttiker formalism, which considers both finite thermal- and disorder-broadening effects [7]. The experimental data are consistent with this formalism, as shown by the fitting in the inset of Fig. 1d (for details, see Methods). The temperature modifies the Fermi–Dirac distribution and, consequently, the occupations of electronic states near the Fermi energy level, causing the temperature-dependent behavior shown in Fig. 1e. Therefore, we attribute the multiple conductance plateaus here to successive subband occupations, which also successfully demonstrates quasi-ballistic transport realization (for low *T*_i_) in A-CNT films.

Quasi-ballistic transport realization can also be characterized by the temperature evolution of the current. The transfer curves of the same device at various temperatures as a function of *V*_g_−*V*_th_ (where *V*_th_ is the threshold voltage) are shown in the inset of Fig. 1f, to eliminate the effects of trapped charges in the gate stack, detailed in Fig. S10. We extracted the currents at the same *V*_g_−*V*_th_ for a temperature range from room temperature to 2 K, the results of which are summarized in Fig. 1f. The current increases with decreasing temperature from 300 to 200 K but slowly decreases below 200 K. The results at high temperatures are associated with suppressed electron‒phonon scattering in diffusive metals. At lower temperatures, thermal excitation through the Schottky barriers at the contacts dominates, leading to a decrease in the current with temperature. Notably, the presence of Schottky barriers does not prevent ballistic transport realization, apart from the reduction in the transmission due to carrier reflections at the contacts, since ballistic transport is only related to scattering in the channel. Furthermore, the current experiences a 4-fold increase when the temperature is reduced from 300 to 2 K, demonstrating suppressed scattering at low temperatures. Considering the low degree of disorders in our devices, the significantly distinct current‒temperature dependencies distinguish the diffusive and quasi-ballistic regimes in different temperature ranges. Consequently, the device here works in the quasi-ballistic regime at low temperatures, although there is a non-perfect transmission at contacts.

### Nonlinear transport spectroscopy of subband occupations

**Figure 2.**
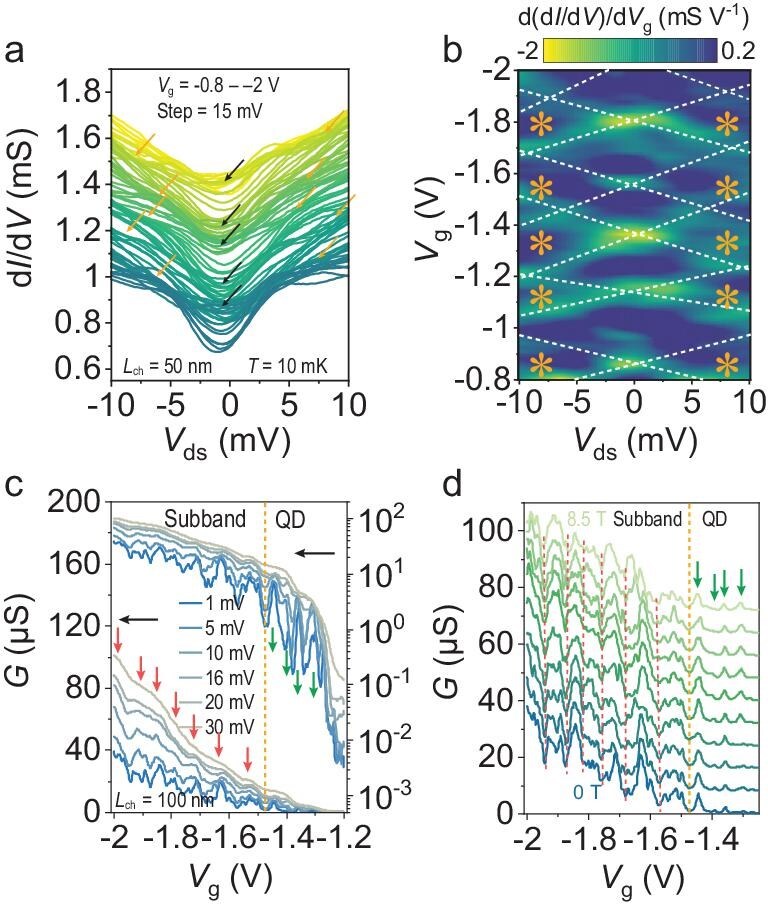
Finite bias spectroscopy of the A-CNT device. (a) Differential conductance of a device with *L*_ch_ = 50 nm as a function of *V*_ds_ at 10 mK for different values of *V*_g_ in the range from −0.8 to −2 V separated by an interval of 15 mV. The black and orange arrows indicate the conductance plateaus at zero bias and the half-plateaus at high bias, respectively. (b) Color scale plot of the transconductance as a function of *V*_ds_ and *V*_g_. The dashed lines are guides for the eye. The orange asterisks mark the half-plateaus at high bias. (c) Transfer curves under various biases at 1.8 K plotted on linear and semilogarithmic scales. The orange dotted line (−1.47 V) distinguishes the subband and QD transport regimes. The red and green arrows indicate the characteristics of the step-like subband and peak-like QD, respectively. (d) Transfer curves at *V*_ds_ = 0.1 mV of the same device as in (c) for various magnetic fields ranging from 0 to 8.5 T. The red dotted lines indicate the positions of the opening of the subbands.

After characterizing the quasi-ballistic transport of the A-CNT films, we further verified the quasi-ballistic transport and extracted the subband separation by performing bias spectroscopy measurements on a device with *L*_ch_ = 50 nm at 10 mK. A series of differential conductance d*I*/d*V* curves as a function of the source–drain bias voltage *V*_ds_ at different gate voltages are plotted in Fig. 2a. The curves accumulate at a specific gate voltage near zero bias, as indicated by the black arrows, which is the signal of the conductance plateau in the equilibrium state. Accumulation of the curves also occurs in the nonlinear regime at high bias, which is identified as the half-plateau features and marked by the orange arrows in Fig. 2a. Nonlinear transport can also be analysed by mapping the transconductance obtained from the derivative of d*I*/d*V* as a function of *V*_g_ and *V*_ds_, as shown in Fig. 2b. The development of conductance plateaus in the nonlinear regime is reflected by the diamond-shaped regions of low transconductance (dark blue), with the half-plateau regions being marked by orange asterisks. The observed characteristics in Fig. 2a and b are analogous to those of QPC in 2DEG [35–37] or some ballistic quasi-1D [13,34] systems. Guided by these similarities, we attribute the above observations to the formation of subbands in our system. The half-plateau features at high bias are induced by the asymmetric occupations of the left- and right-moving subbands within the bias window and arise between the gate voltages of the plateaus at zero bias [38,39]. Previous reports of electronic transport with subband occupations have involved only a small number of channels. The subband occupations in A-CNT systems here are striking, considering that the channel consists of hundreds of individual CNTs. The above observations in A-CNT systems require collective and unified subband occupations in many CNTs as the Fermi level shifts. Otherwise, the complex and differentiated subband occupations would mix and destroy the conductance steps, even if the whole system operates in the ballistic regime. This quantized conductance reveals coupling-induced redistribution of the electronic structure in A-CNT films, as discussed below.

**Figure 3.**
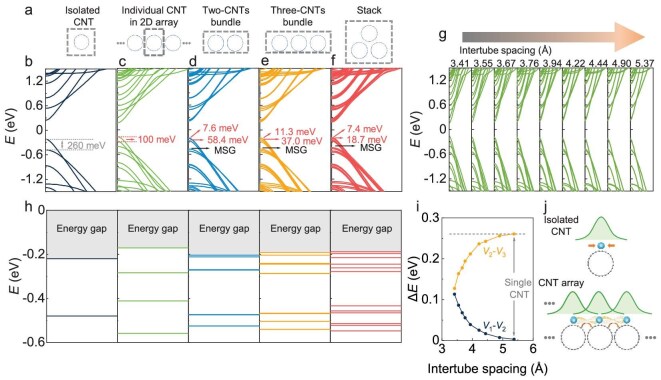
Simulation of the A-CNT system with intertube coupling. (a) Calculated configurations of DFT method used in (b–f). Grey dashed boxes indicate the unit cells or areas. DFT-calculated band structures of an isolated CNT (b), individual CNT among the A-CNT film (c), bundles containing two CNTs (d), and three CNTs (e), and CNT stack (f). All cases used the CNT with chirality (19,0). The energy separations of the first and second subbands are exhibited. The mini-subband gap (MSG) is marked. Intertube spacings in (d–f) are 3.20 Å and that in (c) is 3.41 Å, after geometry optimization. (g) Band structures of individual CNT among A-CNT film with increased intertube spacing, ranging from 3.41 Å to 5.37 Å. (h) Subband distribution of the CNT configurations in (b–f). (i) Subband separations of the first two subbands of the valence band as a function of the intertube spacing, extracted from (g). (j) Schematic illustration of the overlapping wavefunctions and intertube coupling effect in A-CNT films.

We subsequently discuss the energy scale of the subband spacing in A-CNT films to track the effects of intertube coupling. The experimental subband separation ∆*E*_exp_ can be calculated by the carrier concentration in the devices, which is obtained from the integral of the density of states equal to that introduced by the applied gate voltage, based on the expression [40]:


(1) \begin{eqnarray*}
\Delta {E}_{\exp } = \frac{{\pi {\hbar }^2{C}_{\rm ox}}}{{2m^*e}}\Delta {V}_{\rm g},
\end{eqnarray*}

where $\hbar $ is the reduced Planck’s constant, ${C}_{\rm ox}$ is the gate capacitance per unit area, estimated as 1.77 × 10^−2^ F/m^2^ based on the flat capacitance approximation with the relative dielectric constant of HfO_2_ of ∼10 (estimated by the capacitance-voltage measurement in Fig. S11), and m* = 0.12*m*_0_ is the effective hole mass (calculated from the valence band maximum in Fig. 3c). Consequently, the ∆*E*_exp_ values are estimated to be < 10 meV, as shown in Fig. S9b, in agreement with the thermal fluctuation corresponding to the vanishing conductance plateaus in Fig. 1e at 100 K. Moreover, the average energy spacings of ∼5 to 10 meV can be directly obtained from the half-plateau features in the nonlinear regime in Fig. 2b. Similar plateau features also arise in the devices with various channel widths ranging from 560 to 60 nm, as shown in Figs S12 and S13, where plateau features are all smoothed out at ∼100 K, demonstrating the pervasive and robust results with a similar energy scale in A-CNT film. The subband separation in an individual CNT is inversely proportional to its diameter because of the radial quantization condition. For a semiconducting CNT with a diameter near 1.4 nm, the subband separations are ∼0.27 to 0.5 eV [32,41]. Anomalously, the subband separations of the A-CNT film are orders of magnitude smaller than that of an individual CNT. In contrast to the A-CNT films, no plateau-like feature is observed at low temperature and low bias on the transfer curves of the devices based on network-CNT films (Fig. S14), composed of a large number of randomly distributed CNTs and produced by the same arc-discharge CNT powder and purification process without aligned arrangement. The formation of subbands originates from the transverse quantization of the electron wavefunction in quasi-1D systems, whereas continuous energy bands are formed in 2D systems. Consequently, the electronic structure of A-CNT systems cannot be considered simply a superposition of individual CNTs. Here, we propose that the intertube coupling gives rise to a redistribution of subbands in a quasi-2D A-CNT system with parallel arrangement that significantly distinguishes it from 1D and continuous 2D systems.

### Spatial scale of the intertube coupling

First, we begin from the perspective of QPC in a 2D system to discuss the effects of intertube coupling. The expression of the quantized conductance within the QPC formalism is [35]


(2) \begin{eqnarray*}
G = \frac{{4{e}^2}}{h} \times \frac{{{k}_{\rm F}{W}_{\rm ef\!f - QPC}}}{\pi } \times {T}_i,
\end{eqnarray*}

where *k*_F_ is the Fermi wavenumber, *W*_eff-QPC_ is the effective width in the QPC system, and 4 is associated with the valley and spin degeneracies [14,16]. The Fermi wavenumber ${k}_{\rm F} = \sqrt {\pi n} $ is determined by the density of charge carriers *n. T*_i_ is estimated from the first quadruple-degeneracy subband based on an isolated CNT device to be in the range of ∼0.06 to 0.08 (red dashed line in Fig. S15). In the A-CNT system, the maximum number of effective channels can be obtained from the fitting ${N}_{\rm ef\!f - max} = \frac{{{k}_{\rm F - max} \times {W}_{\rm ef\!f - QPC}}}{\pi } = {k}_{\rm F - max} \times \textit{slope} \times \frac{h}{{4{e}^2{T}_i}}$, where *slope* is obtained from the fitting of Fig. S16. The estimated *N*_eff-max_ ranges from 118 to 88 channels and the effective width of the intertube coupling *W*_eff_ *= N_eff-max_ ×* (*d*_CNT_ *+ L*_inter_) = 162–241 nm can be obtained, with an average diameter of *d*_CNT_ ≈ 1.5 nm and intertube distance of *L*_inter_ ≈ 0.34–0.54 nm in the presence of subband redistribution extracted from the DFT calculation (discussed in Fig. 3g), corresponding to the equivalent density is 490–543 CNTs/μm. Second, ∼140 to 105 individual CNTs contribute to the transport in the device, which is extracted from *N*_CNT_ = *G*_max_/(4*e*^2^/*h* × *T*_i_) with the maximum conductance (1.29 mS) in Fig. S16 within the range of the measured gate voltage. When we directly consider the width-dependent number of CNTs, an effective width of ∼193 to 286 nm is obtained. Third, the constricted width of 330 to 165 nm can also be calculated from the energy separation ${\mathrm{\Delta }}E = \frac{{\hbar {v}_{\rm F\pi} }}{{{W}_{\rm ef\!f}}} = 5{-} 10\ {\rm meV}$ which is extracted from the nonlinear transport spectroscopy results in Fig. 2b [34]. Similar *W*_eff_ values are obtained from all the different analyses above. However, the semiquantitative analysis above considers only the situation without interchannel interaction. The scattering induced by interchannel coupling and the overestimated transmission of the A-CNT film at contacts can lead to an underestimation of the effective width [42]. Consequently, we obtain the equivalence of quasi-2D A-CNT films to continuous 2D systems with an effective width.

In addition to subband occupations in the on-state regime, the device can work in the QD regime when tuned by the gate voltage, in which we can also estimate the spatial scale of the intertube coupling. The charge effect is more pronounced in short-channel devices because of the geometrically localized QDs. The differential conductance as a function of the gate voltage and bias voltage is shown in Fig. S17 based on the same device as that used to obtain the results in Fig. 2a and b. Several well-defined Coulomb diamonds can be identified in the pinch-off regime, originating from resonant tunnelling through the QDs. Recognizably, the blockade region of the large diamonds contains additional high-conductance features corresponding to several small diamonds, namely, the coexistence of QDs. The different sizes of the diamonds are related to the various sizes of the QDs in a quasi-2D A-CNT device, which are arranged in parallel in the device channel [43,44]. In this case, intertube coupling leads to the coherence of multiple CNTs and thus the formation of QDs of different sizes. The charge energies of different diamonds range from 11.5 to 2.1 meV. Thus, the lateral extension of the QDs can be calculated to be ∼1 to 7 nm at a channel length of 50 nm based on the geometric area of the QD capacitance (for details, see Supplementary Texts 2). The actual length of the QDs is smaller than the channel length because of the extension of the contact barrier, which leads to a wider geometric length. Therefore, the electronic wavefunctions can be extended to multiple CNTs with intertube coupling in the QD regime, although the intertube coupling is weakened compared with that in the subband regime. The above discussions from subband occupation in the on-state regime to localized QDs in the off-state regime imply the formation of mesoscopic collective coherence in A-CNT systems.

The continuous transition between these two transport regimes can be observed and distinguished based on the evolution of the transfer curves under various bias voltages, as shown in Fig. 2c. A peak-like conductance arises above −1.47 V (marked by green arrows), and a step-like conductance appears below −1.47 V (marked by red arrows), which are related to resonance tunnelling of the QDs and subband occupation, respectively. Apart from the robust subband occupation, there are also some conductance oscillations on the plateaus at a bias of < 10 mV, which originate from the interference effect in the channel or discrete states and demonstrate the realization of ballistic transport and small barriers at the contacts. The same behavior also appears in the device with *L*_ch_ = 50 nm (Fig. S18). The subband occupation characteristics are slightly sensitive to the magnetic field in Fig. 2d because of the small *g*-factor of the CNTs and the disorder broadening effects. The magnetic field dependence of the discrete levels in the QD regime can be used to accurately obtain the *g*-factors of the A-CNT films, which are close to those of isolated CNTs, as shown in Fig. S19 [19].

### DFT simulation of the A-CNT system

To demonstrate intertube-coupling-driven subband redistribution, we simulated the A-CNT system based on the DFT method in Fig. 3. A quasi-2D A-CNT was constructed by (19,0) individual CNTs (with a diameter of 1.5 nm, similar to the situation in the experiment, and high structural symmetry to facilitate the calculations) periodically arranged in a 2D plane with an intertube spacing of 3.41 Å to simulate the A-CNT films. The calculated configurations are indicated by the grey dashed boxes in Fig. 3a (for details of calculation, see Supplementary Texts 4). The calculated band structure is shown in Fig. 3c. A comparison of the band structures of an isolated CNT (Fig. 3b) and an individual CNT in a quasi-2D A-CNT film (Fig. 3c) reveals a substantial increase in the number of subbands and a decrease in the energy separation of subbands in the latter, which is qualitatively consistent with the experimental observations. Furthermore, we changed the intertube spacing of CNTs in Fig. 3c ranging from 3.41 to 5.37 Å, to study the coupling effects since the spacing is directly related to the strength of the intertube coupling (for details, see Supplementary Texts 4). The subband clusters constrict as the spacing increases, which is equivalent to weaker coupling, and are eventually restored to those of an isolated CNT or CNT arrays without coupling, as shown in Fig. 3g and i. Consequently, the above simulations directly demonstrate the coupling-driven redistribution of subband separation, as well as the difference from an isolated CNT and an uncoupled array.

The configurations of the bundles consisting of two- and three-(19,0) CNTs are calculated to discuss the band structure of the whole A-CNT film composed of a large number of CNTs, due to the difficulty of directly calculating the structure of A-CNT films originating from the finite computing performance. Figure 3d and e shows the results of CNT bundles with an intertube spacing of 3.2 Å and AB_2_ lattice alignment after geometry optimization in Fig. S22, which exhibits smaller subband separations than those in Fig. 3b and c, and closes to the experimental observations. The subband distributions in the valence band are summarized in Fig. 3h. Considering the complex morphology and multichiral distribution of the A-CNT film [31], more configurations are calculated, including the stack in Fig. 3f and the bundles constructed by two CNTs with various diameters, chiralities, and alignment configurations, ranging from 1.37 to 1.75 nm (band structure of the isolated CNT is shown in Fig. S23), demonstrated in Figs S24 and S25. Consequently, coupling-driven redistribution of subband separation can be clearly observed in all configurations with different band structures compared with the directed superposition, demonstrating that the phenomenon may mainly arise from the broken symmetry at small intertube spacing, while weakly associated with the diameter or chirality. Notably, the subbands with similar energy of different CNTs in the bundles cause more pronounced band splitting as shown in Figs S24 and S25. Moreover, the subbands become denser and the average separation is smaller as the number of CNTs in the bundle is increased, as shown in Fig. 3h. Hence, band degeneracy is hardly present in the A-CNT film containing a large number of coupled CNTs, and further reduction of average subband separation and denser distributions are expected. An enhanced effect of intertube coupling may emerge in a CNT array with single chirality, giving rise to novel phenomena, such as a recent work by Zhang *et al.* [45]. Additionally, a large separation occurs between the sets of small subband separations, which is defined as a mini-subband gap (MSG). Similar MSG characteristics with higher energy scales are also experimentally observed in Fig. S26. The above physical diagram is described as schematically shown in Fig. 3j. When an array is formed with a large CNT spacing, the characteristics of such an uncoupled array are similar to those of an isolated CNT apart from the increased band degeneracy. As the spacing decreases, the intertube coupling begins to affect the electronic structure of the coupled array due to the overlap of wavefunctions of neighboring CNTs; thus, subband redistribution occurs due to lifting of the band degeneracy by other coupled CNTs. Therefore, the consistency of the experiments and simulations verifies the role of coupling correlations in multichannel systems at the subband level.

### Fano interference between coupled quantum states within a CNT film

**Figure 4.**
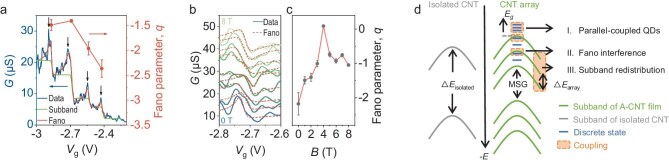
Fano interference and quantum coherence. (a) Transfer curve of a device based on low-density A-CNT films with *L*_ch_ = 400 nm at *V*_ds_ = 1 mV and *T* = 1.8 K. The black arrows represent the subband occupation positions, and the green line represents the fit of the Landauer–Büttiker formalism with the *c* of 0.7 meV. The red lines are fits of the Fano formula, and the Fano parameter *q* values obtained from the fitting are shown by the red lines with dots. (b) Fano shapes at *V*_ds_ = 0.1 mV for various magnetic fields ranging from 0 to 8 T. The dashed red lines show the Fano fitting. (c) Fano parameter *q* obtained from the fitting of the data in (b) as a function of *B*. (d) Schematic band diagram of A-CNT films compared with that of an isolated CNT, showing the coupling effect between the subband and discrete states.

We further investigated the quantum interference between continuous subbands and discrete charge states in low-density A-CNT films. A similar subband occupation feature is also observed in a device based on low-density (∼250 CNTs/μm) A-CNT films, as shown in Fig. 4a. From the fitting of the Landauer–Büttiker formalism, the extracted subband separations are > 11 meV, which is significantly greater than that of the high-density A-CNT device as shown in Fig. S27. The discrepancy in the subband separations demonstrates the redistribution effect of intertube coupling on the band structure of A-CNT films, as discussed above. This originates from the average intertube spacing as well as the morphology, as shown in the cross-sectional TEM images of the A-CNT films with different densities (Fig. S28). The smaller number of CNTs in low-density A-CNT film induces prevalently CNT groups with an inter-group gap and thus the intertube coupling is limited by the group width leading to a large subband separation, while CNTs favor a monolayer arrangement containing few gaps between the CNT groups with a large number of CNTs in the high-density case resulting in a large coupling width and small subband separation. Additionally, we discuss the room-temperature observations of intertube coupling effects calculated by DFT in Fig. 3. The redshifts of *S*_11_ can be observed in the absorption spectra of various CNT systems as shown in Fig. S29, which can be attributed to the changes of band gap induced by coherent tunnelling coupling or modifications of dielectric screening originating from electrodynamic coupling (for details, see Supplementary Texts 5). Considering both thermal fluctuation and realistic CNT systems, the coherence of intertube coupling is destroyed at room temperature and thus no significant reduction of the band gap is observed at room temperature.

Notably, a large overshoot in the conductance appears at the edge of the subband instead of conductance plateaus in Fig. 4a, which demonstrates an additional transmission contribution near the subband edges. The overshooting conductance exhibits asymmetric line shapes. In A-CNT films, the different field efficiencies stemming from the chiral distribution or stacking of CNTs can lead to discrete states, analogous to the formation of QDs in the off-state regime discussed above, whereas most CNTs operate as continuous channels in the subband/on-state regime [46]. Considering the multichannel configuration here, the observed asymmetric characteristics can be ascribed to the Fano interference between coexisting continuous and discrete states in A-CNT systems [47]. Quantitatively, the Fano interference is described by [48]


(3) \begin{eqnarray*}
G &=& {G}_{\rm bg} + {G}_{{\rm Fano}} \times \frac{{{{( \in + q)}}^2}}{{{ \in }^2 + 1}},\\
&&{\mathrm{with}} \in\, = \frac{{\alpha ({V}_{\rm g} - {V}_0)}}{{\Gamma /2}},
\end{eqnarray*}

where *G*_bg_ and *G*_Fano_ are the incoherent and coherent contributions to the conductance, α is the gate efficiency factor, and *V*_0_ and Γ are the position and width of the Fano interference, respectively. The Fano parameter *q* denotes the coupling strength between the continuous and discrete states and affects the asymmetry of the line shapes. The Fano interference is further identified by consistent fitting of equation (3) to the experimental data, as shown in Fig. 4a.

The shape of the Fano interference line can be modified by the coupling strength or the coherent phase difference between the continuous and discrete states, such as by adjusting the gate voltage and magnetic field [49–53]. The red dotted line in Fig. 4a summarizes the Fano parameter *q* as a function of *V*_g_, extracted from fitting with the Fano formula, where |*q*| decreases with decreasing gate voltage. The localization of the discrete states in the channel is weakened in the on-state regime, resulting in a reduction in the Fano coupling strength. In addition, Fig. 4b displays the evolution of Fano interference under various magnetic fields up to 8 T, and *q* as a function of the magnetic field is shown in Fig. 4c. The Fano shape changes with the varied field; in particular, it transitions to a dip at 4 T. Correspondingly, with increasing magnetic field, |*q*| decreases and then increases at 4 T, similar to the observation of modulated phase differences in other nanostructures [49–52]. However, the finite coherent area of Fano interference in A-CNT systems and the small *g* factor result in incomplete observation of the period within the measured magnetic fields. Specifically, the observation and modulation of Fano interference provide further evidence for quantum coherent transport between quantum states in quasi-2D A-CNT systems.

### Quantum coherence in multichannel coupled systems

Finally, we summarize the above observations in different transport regimes, as illustrated in Fig. 4d. Coherent coupling between different states requires the proximity of both their wavefunctions in real space and energy. In the strong pinch-off region (within the energy gap), most CNTs contribute discrete QD states, leading to the formation of parallel-coupled QDs, as shown in regime I. Subsequently, they begin to enter subband transport with a shifting Fermi level, but some discrete states remain due to differences in the gating efficiency, whose coherence gives rise to the Fano interference in regime II. Some discrete levels are not coherent with the subbands because they are far away on the spatial or energetic scale; thus, they behave as small oscillations on the plateau with a smaller energy scale [54]. Finally, subband redistribution occurs due to strong coupling in the on-state regime, as shown in regime III. The larger separation between individual-CNT subbands is defined as the MSG. This physical scenario can be expected to generally describe the quantum coherent transport in multichannel coupled systems.

## DISCUSSION

We have reported collective quantum coherence induced by the characteristic energy scale emerging in artificial A-CNT films, which is prominently distinguished from natural 1D or continuous 2D electronic systems. Using these highly tunable CNT arrays, we explore a new approach to construct an artificially assembled 2D system and observe a characteristic coupling-driven subband redistribution. Our simulation confirms that the band structure of the A-CNT film can be effectively modulated by the intertube coupling. Building on a deep and systematic understanding of the quantum coherence of multichannel coupled systems, high-quality A-CNT films offer a potential future route towards realizing artificial 2D crystals constructed by a long-range periodicity formed in the direction perpendicular to the axial direction of the CNTs [45]. Along with the engineering advantages [28] and controllable intertube coupling [29], the physical properties of A-CNT crystals can be modulated by artificial processes such as lifting speed and concentration of the CNT solution, thus facilitating the development of outstanding electronic and quantum devices with customized electronic structures and versatile functionalities [10,11,25].

## MATERIALS AND METHODS

### Fabrication of a top-gate A-CNT FET

First, marks were patterned via an electron beam lithography (EBL) process, and a 5/90 nm Ti/Au film was deposited via electron beam evaporation (EBE), followed by a standard lift-off process. Second, the channel area was defined by EBL, followed by reactive ion etching (RIE) to remove unnecessary CNTs. Third, contacts were formed by EBL and deposition of 40-nm Pd films via EBE. Next, the gate stack was patterned via EBL, and the gate dielectric of an HfO_2_ film was deposited via atomic layer deposition (ALD). Thermal ALD growth was performed at 90°C using a Beneq TFS-200 system, with a tetrakis(dimethyl amido) hafnium (IV) (TDMAH) precursor and an H_2_O oxidizer. The optimized method used to improve the CNT/HfO_2_ interface [30], that 0.25 s H_2_O pulse/25 s N_2_ purge repeated for 20 cycles, before HfO_2_ growth with the process 0.5 s TDMAH pulse/25 s N_2_ purge/0.5 s H_2_O pulse/25 s N_2_ purge repeated for 42 cycles (about 5 nm). Afterwards, a 30-nm Pd film was continuously deposited as a gate metal to reduce exposure of the interface to the atmosphere. Finally, 5/90-nm Ti/Au bilayer films were patterned for connection and pads.

### Electrical measurement

The as-fabricated devices were measured in a physical property measurement system (Quantum Design DynaCool) with a base temperature of ∼1.8 K and a maximum magnetic field of 8.5 T. The results at 10 mK were obtained in a cryogen-free dilution refrigerator. The bias and gate voltages were applied with a Yokogawa GS200 source, and the current signal was recorded by a 34401A digital multimeter (Keysight) after passing through an SR570 current preamplifier (Stanford Research). The a.c. lock-in measurement was performed with a low-frequency (17.7 Hz) SR830 lock-in amplifier (Stanford Research).

### Landauer–Büttiker formalism fitting

The generalized Landauer–Büttiker formalism, which considers both finite thermal- and disorder-broadening effects, is given by


\begin{eqnarray*}
G = \frac{{4{e}^2}}{h}\mathop \sum \limits_i \int {T}_i\left( E \right)F\left( E \right)G\left( E \right){\rm d}E,
\end{eqnarray*}

where *F*(*E*) and *G*(*E*) are the thermal and disorder broadening functions, respectively.


\begin{eqnarray*}
F( E) = \frac{1}{{4{k}_{\rm B}T}}{\mathrm{sec}}{{\mathrm{h}}}^2\left( {\frac{{E - {E}_{\rm f}}}{{2{k}_{\rm B}T}}} \right),
\end{eqnarray*}

\begin{eqnarray*}
G( E) = \frac{1}{{c\sqrt {2\pi } }}{\mathrm{exp}}\left( { - \frac{{{{\left( {E - {E}_{\rm f}} \right)}}^2}}{{2{c}^2}}} \right),
\end{eqnarray*}

where *c* is the variance of the Gaussian distribution function. The relationship between *E*_f_ and *V*_g_ is obtained via equation (1).

### Optical experiments

The CNT films arranged on the Si/SiO_2_ substrate were transferred to a quartz substrate based on a poly(methyl methacrylate) (PMMA)-assisted wet transfer method before observing the visible/near-infrared absorption spectrum (measured by Cary 5000). The photoluminescence map of the arc-discharged CNT solution extracted by PCz was measured using a spectrofluorometer (Nanolog, HORIBA). The Raman spectra were measured using a spectrometer (HR800, HORIBA).

## Supplementary Material

nwag052_Supplemental_File
