# Supplementary material for: Collective quantum coherence and subband redistribution in artificially assembled nanotube arrays
Source: Natl Sci Rev. 2026 Jan 27;13(6):nwag052. doi: 10.1093/nsr/nwag052 (PMC13017835; doi:10.1093/nsr/nwag052)
Supplement: nwag052_Supplemental_File [file nwag052_supplemental_file.pdf]

Supplementary Materials for

Collective quantum coherence and subband redistribution in artificially assembled nanotube arrays

Xiao-Song Deng *et al.*

*Corresponding author. E-mail: Ning Kang, nkang@pku.edu.cn; Zhiyong Zhang, zyzhang@pku.edu.cn

This PDF file includes:

Supplementary Texts 1-5

Figures S1 to S32

Table S1

References

Supplementary Texts 1. Fabrication of A-CNT film.

Preparation of a high-purity CNT solution. First, 2 mg of conjugated poly[9-(1-octylnonyl)-9H-carbazole-2,7-diyl] (PCz) and 2 mg of arc-discharged CNT powder (purchased from Carbon Solution, Inc.) were dissolved in 500 mL of toluene. The mixed solution was then dispersed with a 7 mm probe tip for 300 min at 600 W (Sonics VCX-800), and the dispersion was centrifuged at 50 000 × g by a Sorvall LYNX 6000-Thermo for 2 h as follows. The as-prepared PCz-wrapped CNT solution was filtered via a dynamic liquid-phase process, followed by repeated rinsing in 1,4-epoxybutane (THF). The filtered PCz-wrapped CNTs were redispersed in a 1,1,2-trichloroethane target solvent for 5 min at 600 W (Sonics VCX-800). The above dispersion and centrifugation processes were repeated to obtain a high-purity CNT solution. Finally, semiconducting CNT purified solution with the concentration of 80 and 40 ug ml^{-1} is obtained for the preparation of high- and low- density A-CNT films, respectively.

A-CNT arrays on a 4-in. wafer. Initially, 160 mL of PCz-wrapped CNTs in 1,1,2-trichloroethane were dropped into a vessel with a geometric size of L:W:H = 11:1.5:11 cm. A 4-in. high-resistance silicon wafer was immersed in the as-prepared CNT solvent and clamped by a dip-coating mechanical apparatus. To form a binary liquid interface for subsequent CNT deposition onto the wafer, 40 μL of $C_4H_8O_2$ (2-butene-1,4-diol) was subsequently added to the CNT solution. Afterwards, the 4-in. wafer was withdrawn at the speed of 1 μm s^{-1} for high density and 2 μm s^{-1} for low density, and the A-CNT arrays were uniformly distributed throughout the entire 4-in. wafer within 3 h. The A-CNT film was then rinsed with toluene, THF and N,N-dimethylformamide for at least 20 min for each solvent. The deposition was finished by baking the wafer at 180 ℃ for 30 min.

Pretreatment of A-CNT arrays. *Annealing process.* The air in the tube furnace (Thermo Scientific Lingerg/Blue M Moldatherm 1100 ℃) was first blown away with 1000 sccm argon, and the A-CNT wafer was placed in the tube furnace for annealing for 3 h. We set the annealing temperature at 600 ℃, and the argon and hydrogen flow rates were 40 and 5 sccm, respectively. The main purpose of this process was to

remove excess polymer molecules wrapped on the CNTs in the array film.

Yttrium oxide coating and decoating process: First, 5 nm yttrium was deposited on the A-CNT wafer, followed by thermal oxidation at 250 ℃ in an atmospheric environment for 30 min. Afterwards, the yttrium oxide film was removed with 20 times H_2O-diluted hydrochloric acid solution for 20 min, and the wafer was then repeatedly rinsed in DI water and isopropanol.

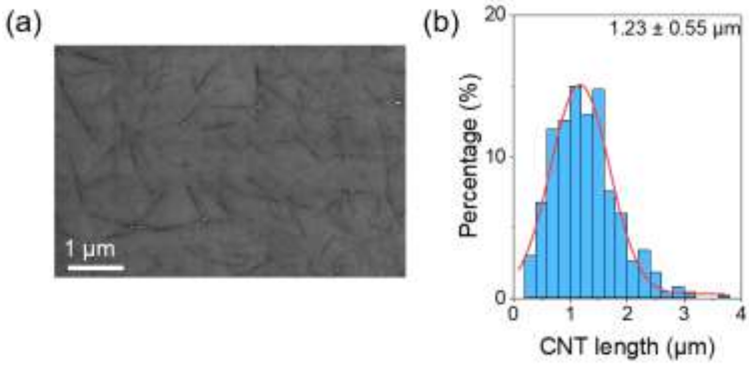

Figure S1. (a) SEM image of randomly-orientated CNTs film using the same CNT solution; (b) The statistics length distribution of CNTs used for A-CNT films.

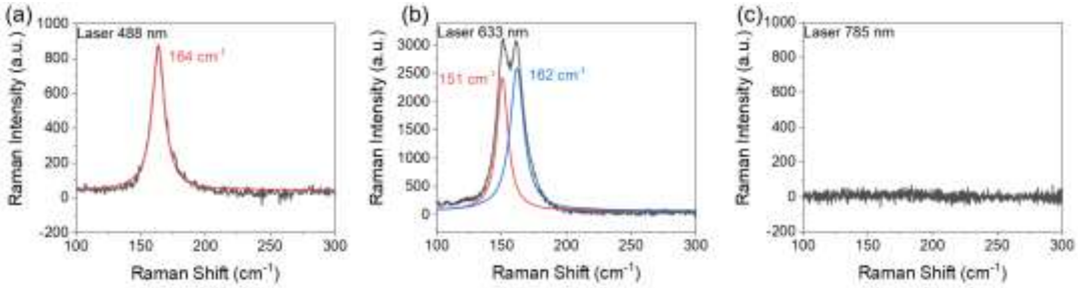

Figure S2. Raman spectrum of A-CNT films on the SiO_2/Si substrate with various laser excitation wavelengths of 488 (a), 633 (b), and 785 nm (c), respectively. Black lines indicate the measured data, and colored lines are the fitting of the radial breathing mode by a Lorentz line shape to obtain the diameter of the CNTs. The diameter of the CNTs in the A-CNT films can be obtained by the peaks of radial breathing mode near 150 cm^{-1}, $\omega_{RBM} = A/d_{CNT}+B$, where A = 248 cm^{-1} and B = 0 cm^{-1} for the single-walled CNT on the SiO_2/Si substrate. [1]

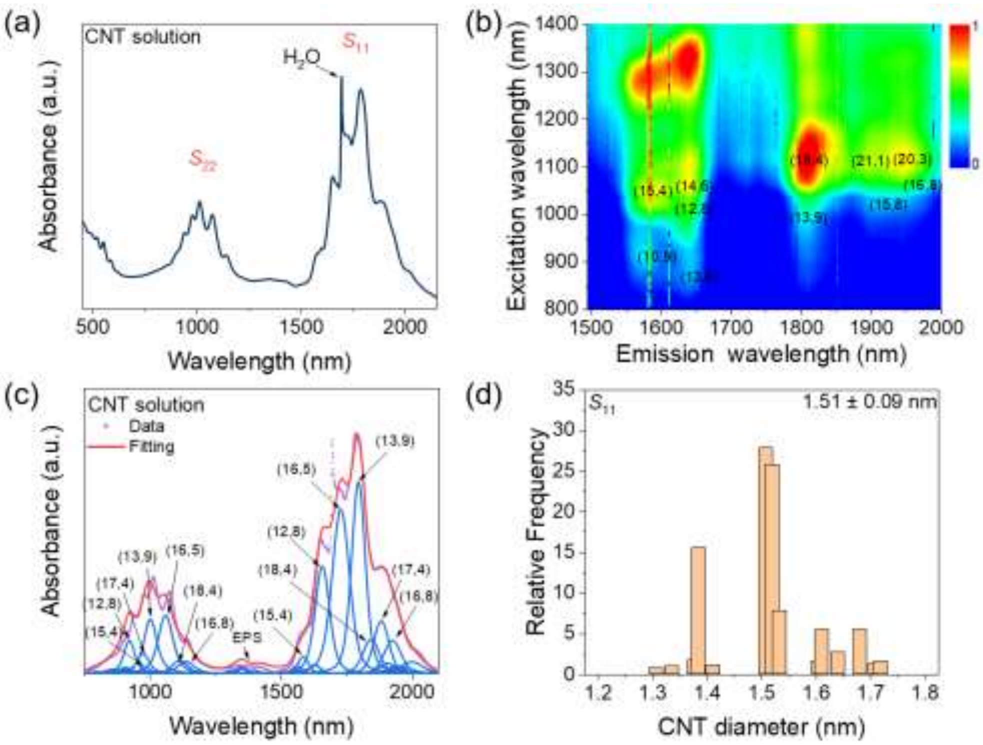

Figure S3. Optical measurement of the arc-CNT solution extracted by PCz. Absorption spectrum **(a)** and photoluminescence map **(b)** of the CNT solution. The absorption peak at ~1690 nm originates from the water molecules unintentionally introduced during solution preparation. **(c)** Fitting of the absorption spectrum after background subtraction. The chiral indices of the CNTs used in the fitting process are all extracted from the photoluminescence map in **(b)** and the Raman spectrum in Fig. S2. The k/λ^b background subtraction, Voigt line profile, and the contribution of exciton-phonon sidebands (EPS) are performed. **(d),** Diameter distribution calculated from the spectral weight of S_{11} in the fitting result of **(c)**. [2]

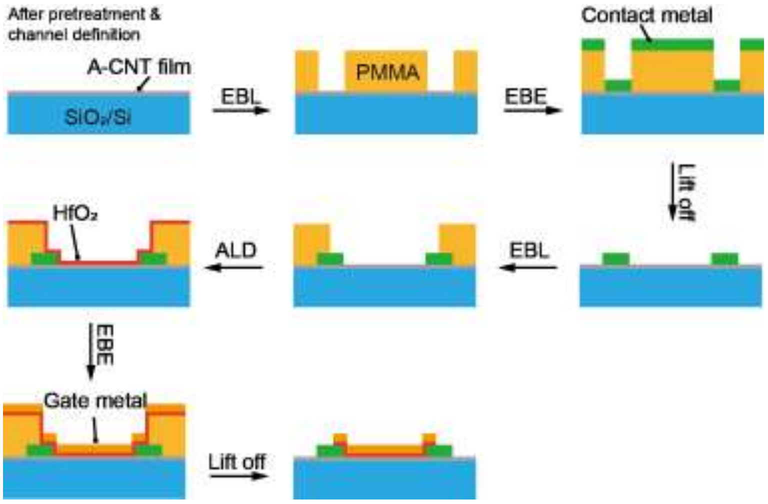

Figure S4. Schematic of the fabrication process of the device based on A-CNT films. First, the contact window is patterned via electron beam lithography (EBL, Raith Voyager) with the E-beam resists of PMMA, after pretreatment and channel definition of A-CNT films. Second, the contact metal Pd of 40 nm is deposited via electron beam

evaporation (EBE, DE400), followed by standard lift-off process with acetone. Third, the same process is conducted to pattern the gate window via EBL. Next, the gate dielectric of a 5 nm (42 cycles) HfO_2 is deposited via ALD (Beneq TFS-200) at 90 ℃. Afterwards, a 30 nm Pd film is continuously deposited as a gate metal via EBE. Finally, a standard lift-off process with acetone is used to form the HfO_2/Pd gate stack.

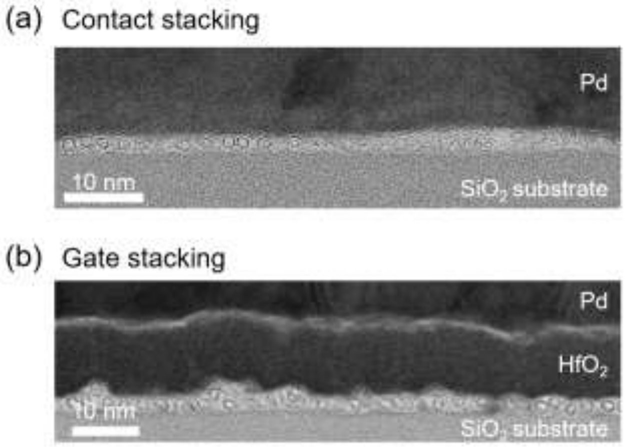

Figure S5. Cross-sectional TEM image of the contact (a) and gate (b) stackings in the devices based on A-CNT films.

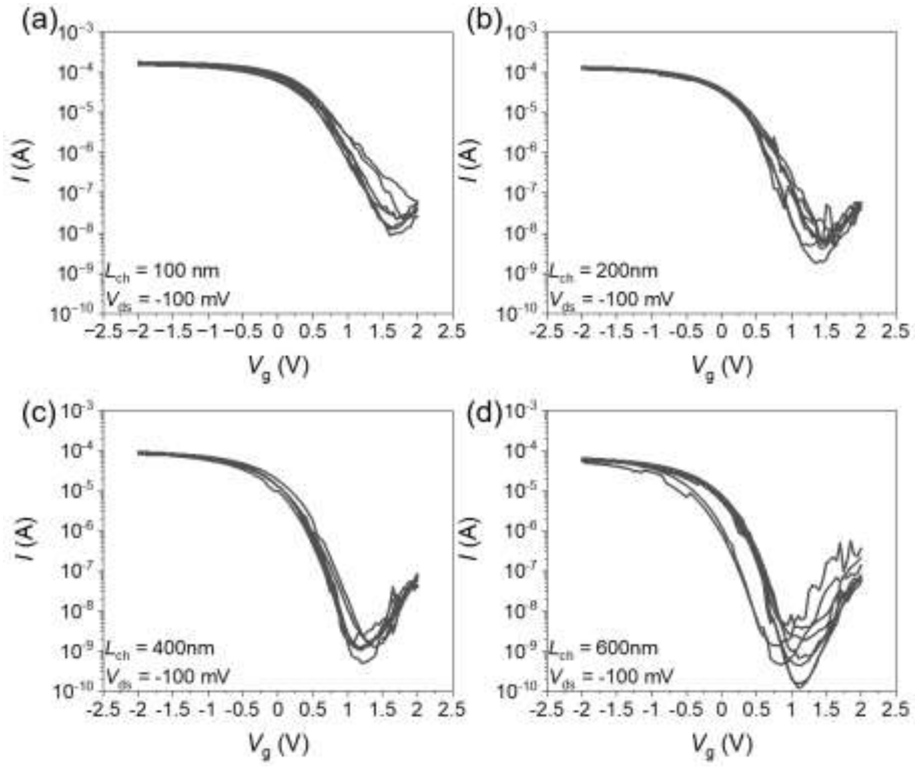

Figure S6. Transfer curves of devices with various channel lengths based on high-density A-CNT films at 300 K. The channel width of all the devices is 800 nm.

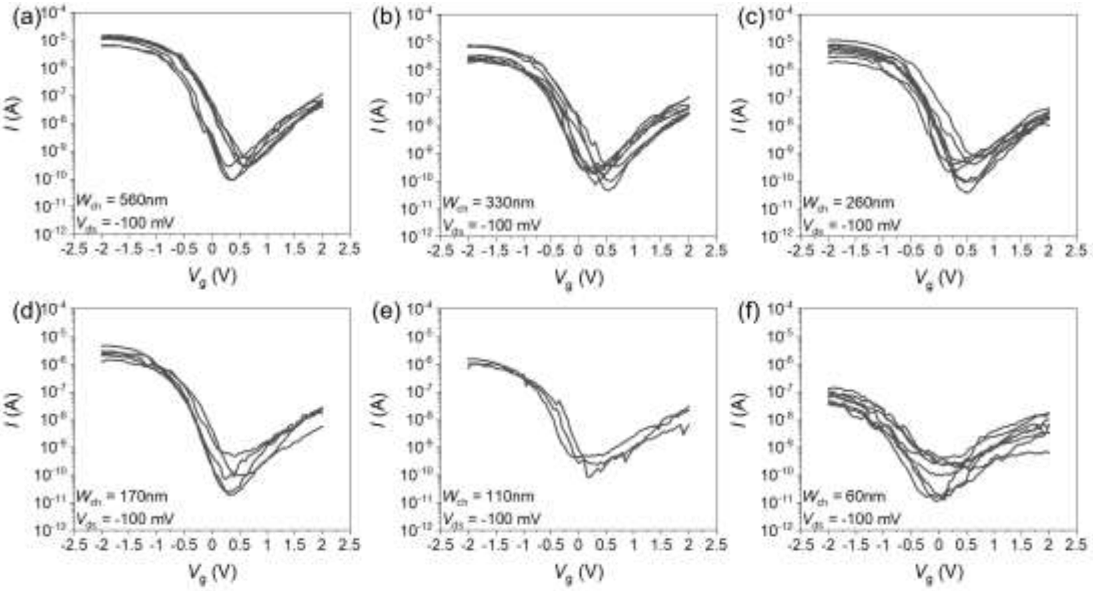

Figure S7. Transfer curves of devices with various channel widths based on high-density A-CNT films at 300 K. The channel length of all the devices is 400 nm.

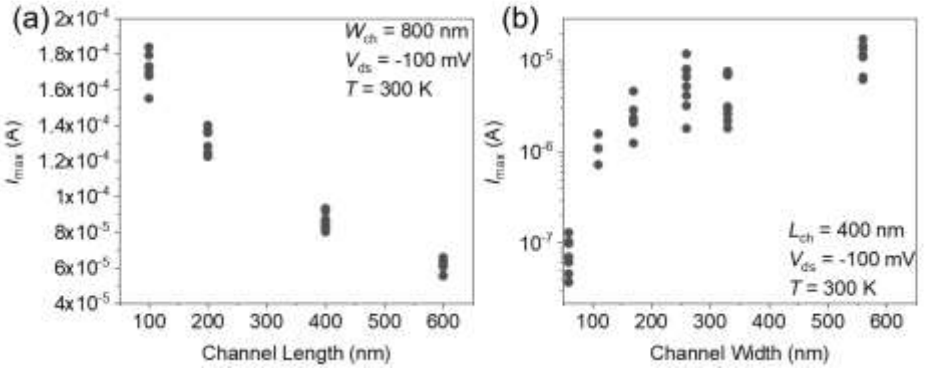

Figure S8. Statistical results of the maximum currents extracted from Figs S6 and S7. The transport in devices with narrow channel widths is more susceptible to non-ideal arrangements, leading to increased fluctuations of transport behavior.

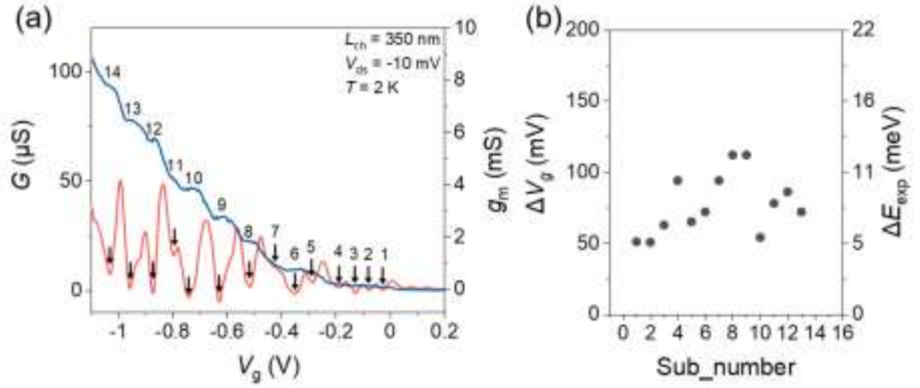

Figure S9. Subband separations of a device based on A-CNT films. (a) A transfer curve and transconductance curve taken at 2 K. The black arrows are the minima of the transconductance and the numbers are the subband orders, indicating the position of

each subband. **(b)** Subband separations as the function of subband number, extracted from **(a)**. ΔE_{exp} is calculated from the ΔV_g by the equation (1). The data from the same device of Fig.1.

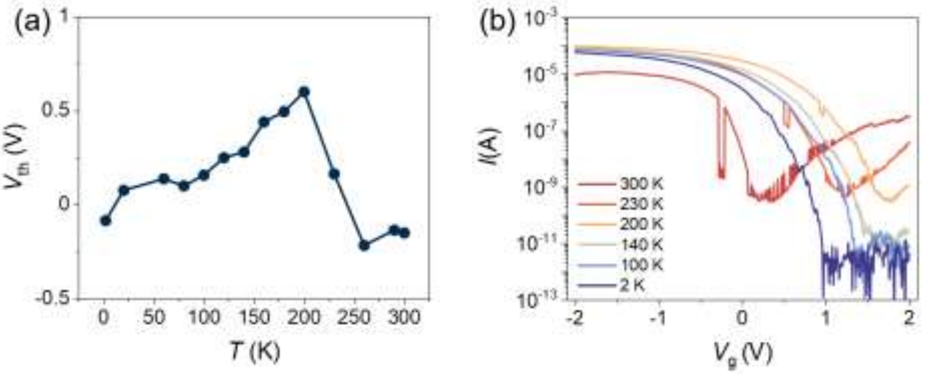

Figure S10. Variation of V_{th} with temperatures. (a) V_{th} as a function of temperature, extracted from the inset of Fig. 1f. **(b)** Raw transfer curves at several temperatures. The current jumps observed near 1 μA at high temperatures are caused by the switching ranges of the measurement equipment. The data are obtained from the same device with Fig 1. The entire transfer curves shift horizontally with temperatures and the current at the neutral point remains unchanged above 200 K, while the current at the neutral point rapidly decreases to the measurement limit with an expanded range of pinch-off regime below 200 K. The former arises from the temperature-dependent redistribution of the trap states in the gate stack, while the latter stems from the significant suppression of thermally excited transport in the subthreshold regime, both lead to variations in the V_{th} with temperatures.

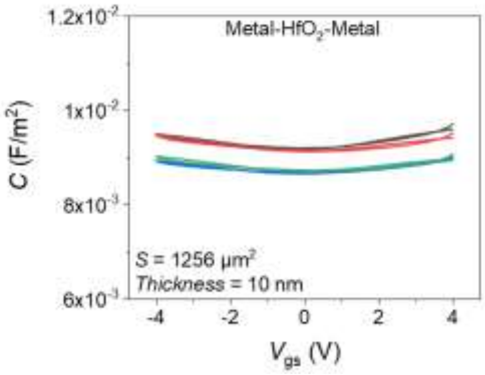

Figure S11. Capacitance-voltage measurement at 1 MHz of the HfO_2 films. Capacitance on a metal-HfO_2-metal structure on a bare silicon wafer with an area of 1256 μm^2 and a thickness of 10 nm. Different colors indicated the different positions of the wafer. $\varepsilon_r \sim 10$ is calculated from the average normalized capacitance of 8.8~9.3 $\times 10^{-3}$ F/m^2 based on the flat capacitance approximation.

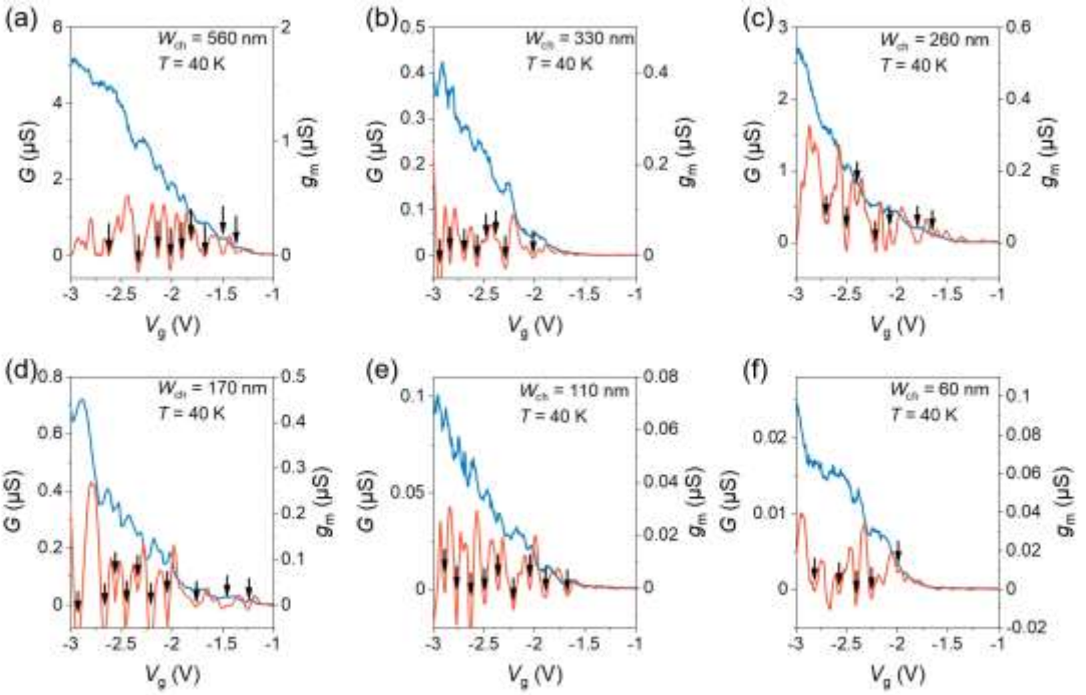

Figure S12. Conductance and transconductance as a function of gate voltages in the devices with various channel widths based on high-density A-CNT films at 40 K. The channel length of all the devices is 350 nm. The plateau-like features are indicated by the black arrows, defined as the dip of transconductance.

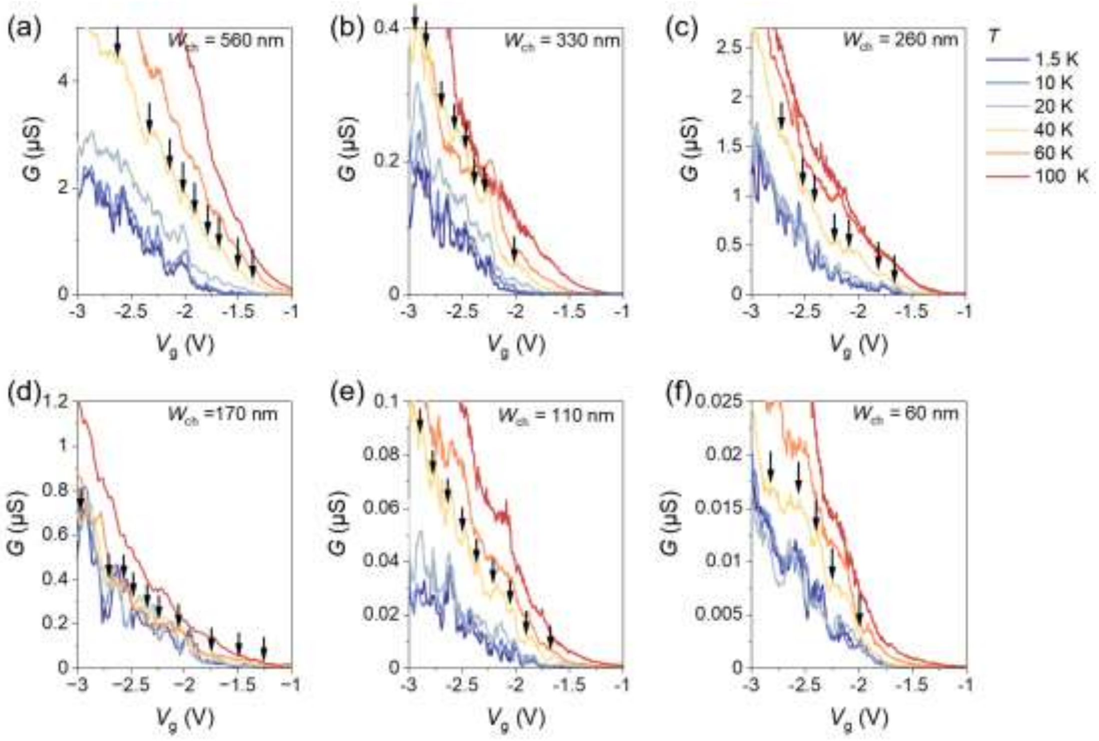

Figure S13. Temperature dependence of the transfer curves in the devices with various channel widths based on high-density A-CNT films. The channel length of all the devices is 350 nm. The plateau-like features are indicated by the black arrows.

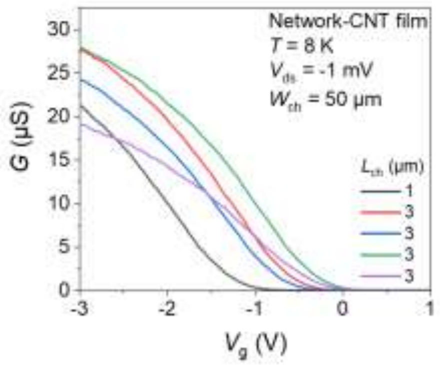

Figure S14. Transfer curves of the network-CNT films-based FET measured at temperature of 8 K and bias voltage of -1 mV. No plateau-like feature is present on the transfer curves, in contrast to the observations in A-CNT films.

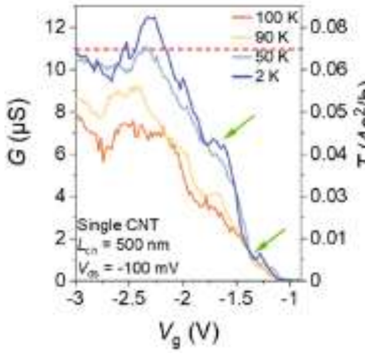

Figure S15. Transfer curves at various temperatures of a device based on an individual CNT. The individual CNT is separated from the A-CNT films. The conductance increases with decreasing temperature, demonstrating the quasi-ballistic transport. Non-perfect contact leads to non-perfect transmission. Red dashed line indicates the conductance plateau corresponding to the first quadruple-degeneracy subband, recognized as the first quadruple-degeneracy subband with the average conductance of G_i = 10~12 μS. The transmission coefficient can be estimated by $T_i = G_i / (4e^2/h)$ = 0.06~0.08. Green arrows indicate the plateau-like feature, originating from the anomalous degeneracy lifting.

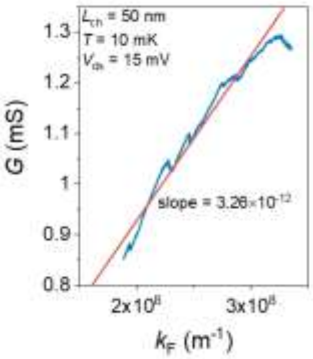

Figure S16. Conductance as a function of the Fermi wave number. The red line is a fit using the *Eq.* (2) in the ballistic regime. The effective width of the constriction can be obtained from the slope of the fitting.

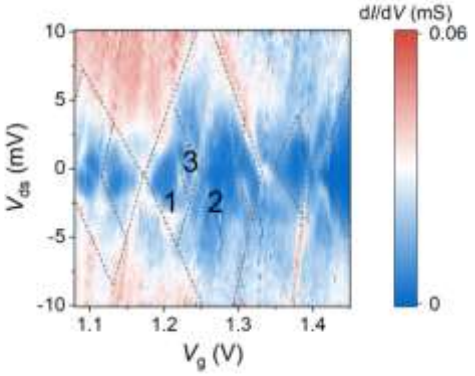

Figure S17. Differential conductance as a function of V_g and V_{ds}, showing the nested diamonds with different sizes. The charge energies change from 2.1 to 11.5 meV.

Supplementary Texts 2. Geometric size of the quantum dot.

The constant-interaction model [3] gives the addition energy, $E_{add} = E_c + \Delta\epsilon$, where $E_c = e^2/C$ is the charge energy, C is the total capacitance and $\Delta\epsilon$ is the energy difference between adjacent levels. It is reasonable to ignore $\Delta\epsilon$ in multi-electron regime. Then, the total capacitance can be obtained from E_c. The gate capacitance $C_g = e/\Delta V_g$ can be extracted from the width of diamonds, where the source/drain parasitic capacitance can be neglected compared with C_{ox} [4]. Finally, the geometric size of the quantum dot is calculated by $S = C_{ox}/C_g \approx L_{ch} \times W_{qd}$, where $C_{ox} = \varepsilon_0\varepsilon_r/t_{ox}$=1.77×$10^{-2}$ F/m^2 by flat capacitance approximation here (ε_0 is the vacuum permittivity, $\varepsilon_r \approx 10$ is the relative permittivity of HfO_2 obtained from the empirical value, and $t_{ox} \approx 5\,nm$ is the thickness of the gate oxide). The parameters of representative diamonds of Figure S17 are summarized in the Table S1.

Table S1 Parameters of representative diamonds in Fig. S17

diamond	ΔV_g (mV)	E_c (meV)	C_g (F)	C (F)	S (nm^2)	W_{qd} (nm)
1	74.7	5.2	$2.14e^{-18}$	$3.01e^{-17}$	121	2.4
2	159.4	11.5	$1.00e^{-18}$	$1.39e^{-17}$	56.7	1.1
3	24.7	2.1	$6.48e^{-18}$	$7.62e^{-17}$	366	7.3

There is an underestimation of W_{qd}, considering that the actual length of QDs is smaller than L_{ch}. Moreover, a greater lateral extension can be obtained by a round shape of QDs. Consequently, we capture the formation of the large sizes of QDs based on multiple-CNT coherence, since the average diameter of CNTs is 1.5 nm. The observation demonstrates that the wave function of the electron in a QD extends to multiple CNTs. The intertube coupling can be controlled by the gate voltage, causing the smaller spatial scale than that in subband regime.

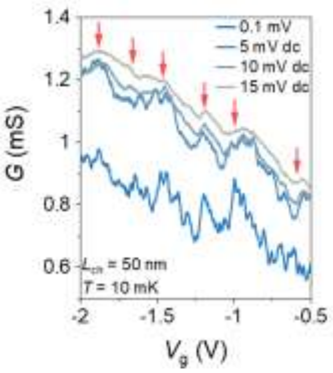

Figure S18. Transfer curves at various biases of a device based on A-CNT films. Red arrows indicate the positions of subband. The curve with 0.1 mV is measured by the lock-in ac technology, while others are measured by the dc technology. Obvious interference appears in the curve with 0.1 mV, demonstrating the ballistic realization.

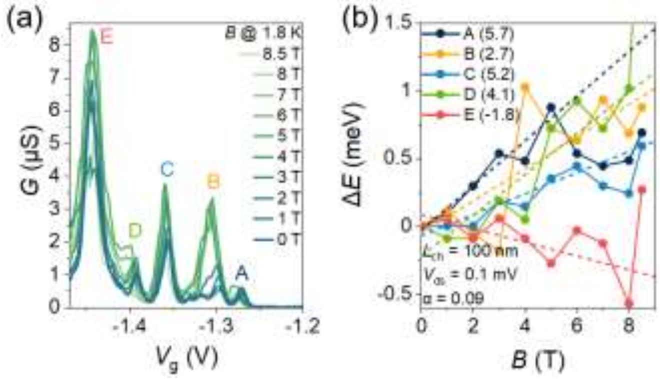

Figure S19. Magnetic field dependence of the discrete levels in the QD regime. (a) Transfer curves as a function of V_{g} at various magnetic fields. Peaks indicate the resonance transport through the discrete levels of the QDs, marked by the colored letters. **(b)** The shifts of the conductance peak in **(a)** as a function of magnetic fields. ΔE is obtained by the equation (1). Dashed lines are the fitting of Zeeman equation (S1).

Supplementary Texts 3. Level shifts induced by magnetic field.

The discrete levels in QD can shift with a perpendicular magnetic field due to the Zeeman effect [5,6]. The contribution of orbital magnetic moment can be neglected without parallel magnetic field. Thus, the Zeeman shift is given by

$$\Delta E_z = \frac{1}{2} g_s \mu_B B_\perp \#(S1)$$

where g_s is the spin g factor and μ_B is the Bohr magneton. We fit the level shifts in Fig. S19a and the results are shown in Fig. S19b g_s is obtained from 1.8 to 5.7, close to that of single CNTs.

Analogously in subband regime, the shift of subband is calculated to be less than 0.5 meV at 8 T, which is close to the disorder broadening with 1.7 meV from the fitting of Landauer-Büttiker formalism in the inset of Fig.1d. Thus, the magnetic field-induced subband shift is masked by disorder broadening and cannot be resolved experimentally. Meanwhile, intertube coupling between adjacent CNTs also lift an amount of subband

degeneracy as discussed in the main text. This reduces the remaining degeneracy that the magnetic field could further lift, weakening the observable effect of the magnetic field on subband characteristics.

Supplementary Texts 4. DFT simulation of A-CNT system

The DFT calculations are performed using ab initio methods as implemented by Vienna *ab initio* Simulation Package (VASP) [7] using the projector augmented wave (PAW) [8]. The electronic exchange-correlation interactions were modeled within the generalized gradient approximation with the Perdew–Burke–Ernzerhof functional (GGA-PBE) [7,8]. The long-range van der Waals interactions were included using the semiempirical DFT-D3 method [9,10]. A plane wave energy cutoff of 500 eV is used with k-meshes of $1\times 1\times 11$ and $3\times 1\times 11$ for 1D CNTs and 2D A-CNTs system, respectively. Both the lattice parameter and atom coordinates are fully relaxed, until the energy is converged at 10^{-6} eV and the force is converged at 0.02 eV/Å for the geometry optimization. The (19,0) CNT is used to model the single CNT with a 1.5 nm diameter similar to the experiment, which lattice parameter is 4.28 Å and the energy band gap is 0.46 eV. The 1D CNTs and 2D A-CNTs system is modeled as a 3D lattice in which the distances of CNTs at lattice aperiodic direction are chosen to be more than 10 Å so that there is negligible interaction between aperiodic CNTs in the periodic lattice. To clarify the change of electron coupling in 2D A-CNTs with the inter-tube interaction, lattice strain was applied at the non-transport direction in array system. The strain-driven changes of intertube spacing and system energy are in Fig. S20. The intertube spacing is defined as the distance between two tubes. The strain energy ($E_{str.}$) is defined as:

$$E_{str.} = E_{tot.} - E_{tot.(str.)}$$

Here, the $E_{tot.}$ is the total energy of 2D A-CNT, $E_{tot.(str.)}$ is the total energy of 2D A-CNT under strain. The strain dependence of band structure is in Fig. S21. In Fig. S21, the E_{vx} is defined as the sequencing of the energy band under fermi energy, example the $E_{v1\text{-}2}$ is the sub energy between the first and second energy. While to evaluate the interactions between the tubes bundle, the two-CNTs bundle and three-CNTs bundle models are established, and the electron levels are shown in Fig. 3d and e.

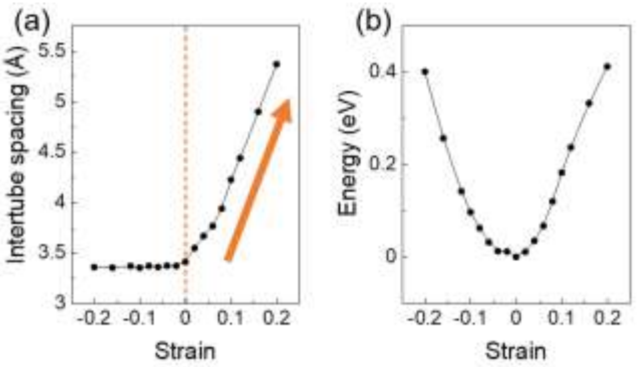

Figure. S20. Strain-driven changes of intertube spacing (a) and system energy (b)

in DFT simulation of A-CNT system.

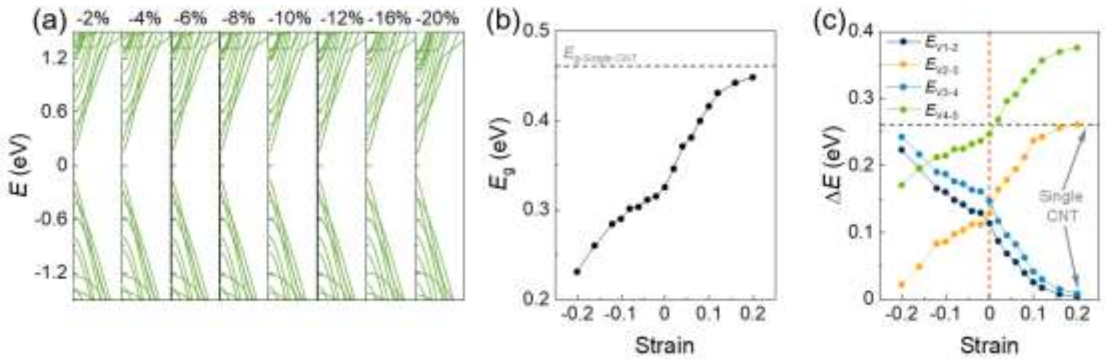

Figure S21. Strain dependence of band structure. (a) Band structures of individual CNT among A-CNT films with negative strain. **(b)** Energy gap of A-CNT films as a function of strain. Dotted line marks the energy gap of ab isolated CNT. **(c)** Subband separations of the first five subbands of the valence band as a function of strain, extracted from Figs 3g and **(a)**. Dotted grey line marks the subband separation of the first subband of isolated CNT.

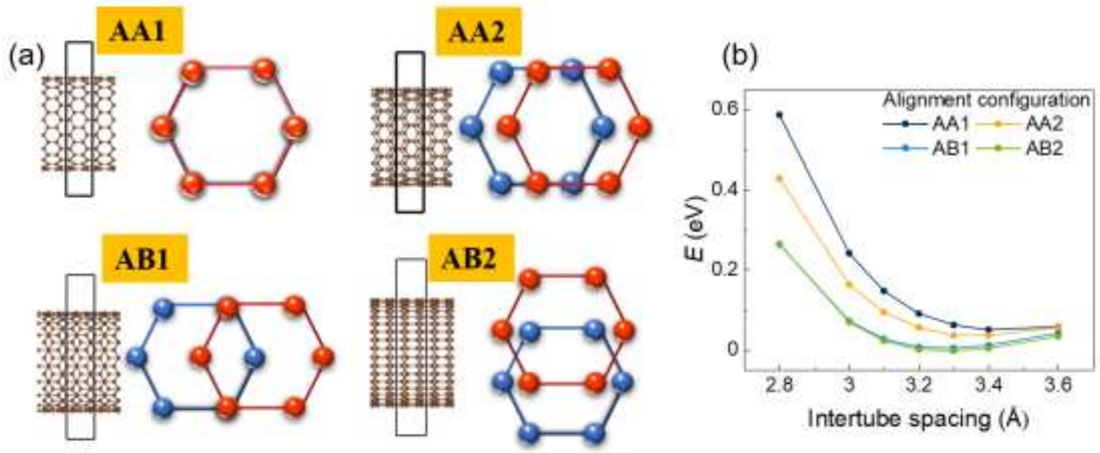

Figure S22. Alignment configurations of zigzag-CNT bundle in DFT calculation. (a) Side views and schematics of the lattice alignment in CNT bundles with various alignment definitions. **(b)** System energy of the (19,0)-CNT bundle as a function of intertube spacing with various alignment configurations. The alignment with lowest energy is AB2, used in Fig. 3d and e.

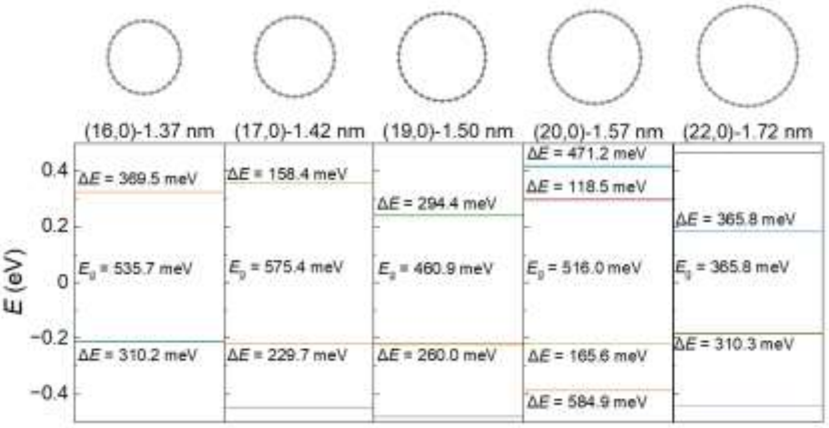

Figure S23. Band structures of the isolated individual CNTs with various diameters, ranging from 1.37 nm to 1.72 nm. Colored lines indicate the subbands. The subband separations, Δ*E*, are indicated in the band structures.

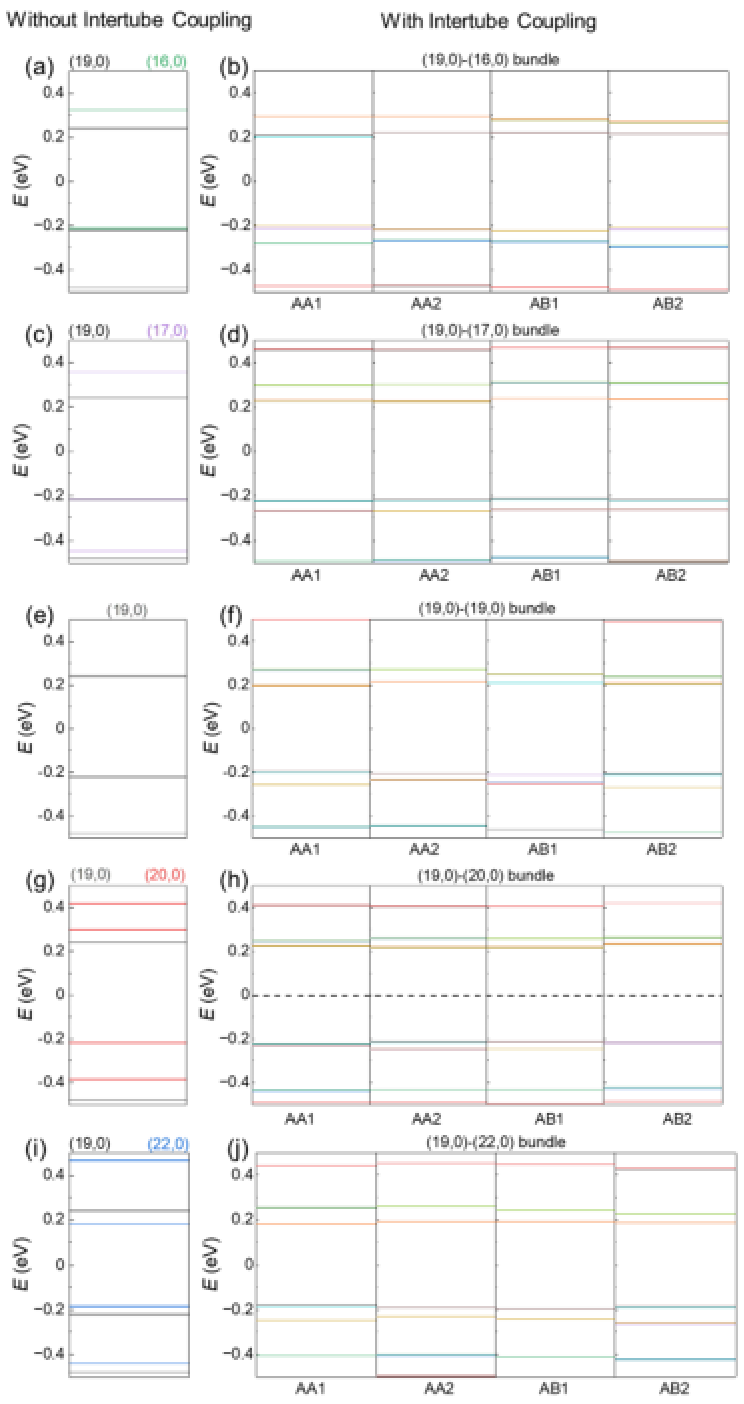

Figure S24. Band structures of bundles constructed by the CNTs with different diameters at various alignment configurations. Left panel: direct superposition of the band between different CNTs, including (19,0)-(16,0) in **(a)**, (19,0)-(17,0) in **(c)**, (19,0)-(19,0) in **(e)**, (19,0)-(20,0) in **(g)**, and (19,0)-(22,0) in **(i)**, respectively, where no intertube coupling exits. Right panel: band structure of the bundle constructed by different CNTs at various alignment configurations, including (19,0)-(16,0) in **(b)**, (19,0)-(17,0) in **(d)**, (19,0)-(19,0) in **(f)**, (19,0)-(20,0) in **(h)**, and (19,0)-(22,0) in **(j)**, respectively. The different band structure between the two cases indicates the effect of intertube coupling, demonstrating the weak relationship of diameters.

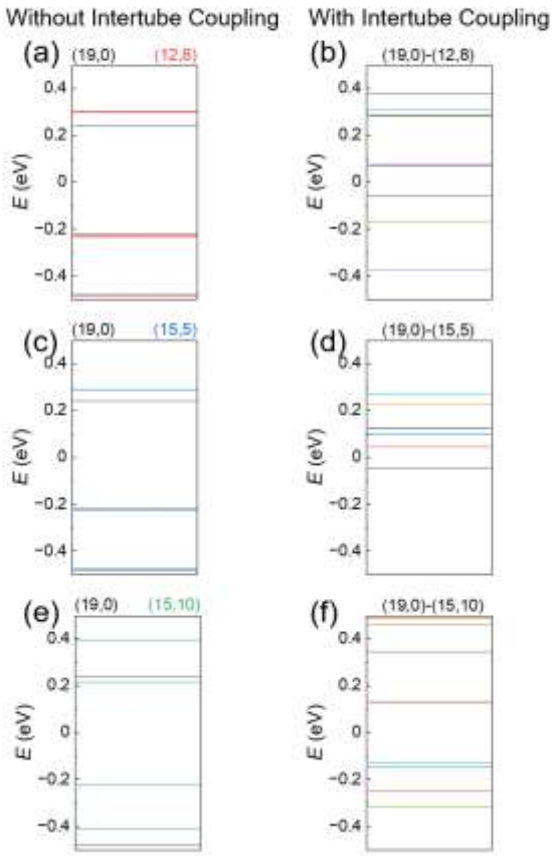

Figure S25. Band structures of bundles constructed by the CNTs with different chiralities. Left panel: direct superposition of the band between different CNTs, including (19,0)-(12,8) in **(a)**, (19,0)-(15,5) in **(c)**, and (19,0)-(15,10) in **(e)**, respectively. Right panel: band structure of the bundle constructed by different CNTs, including (19,0)-(12,8) in **(b)**, (19,0)-(15,5) in **(d)**, and (19,0)-(15,10) in **(f)**, respectively. The different band structure between two cases indicates the effect of intertube coupling, demonstrating the weak relationship of chiralities.

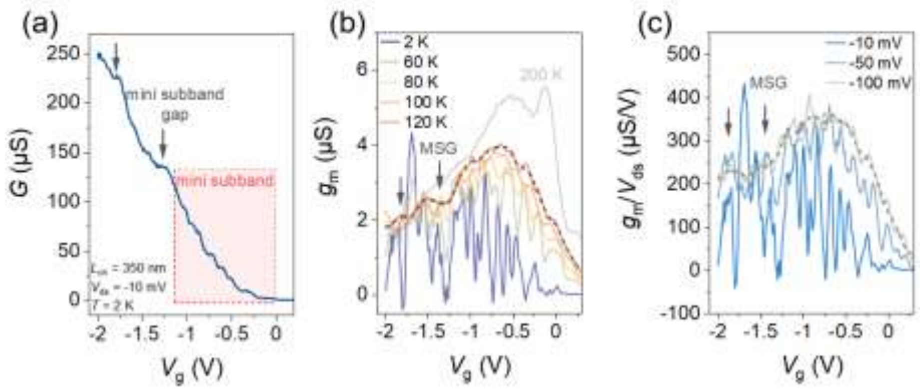

Figure S26. Mini subband gap in a device based on A-CNT films. (a) Transfer curves at 2 K. Black arrows mark the position of mini subband gap (MSG) with a larger energy scale, compared with the normal subband. Red region is the mini subband occupancy shown in Fig. 1d. Temperature **(b)** and bias **(c)** dependence of MSG demonstrate the larger energy scale of MSG. The characteristics of MSG can be sustained up to 200 K or 100 mV after that of mini subband occupancy disappears. The data from the same device of Figure 1.

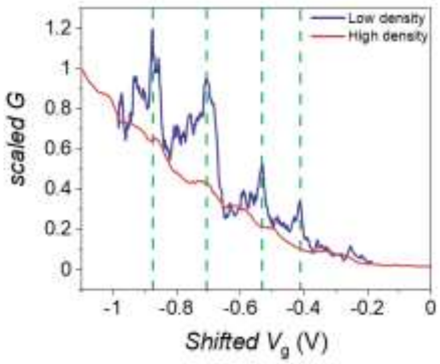

Figure S27. Scaled transfer curve of the devices based on low-density and high-density A-CNT films, obtained from Figs 4a and 2d. Green dashed lines indicate the subband features of the device based on low-density A-CNT films.

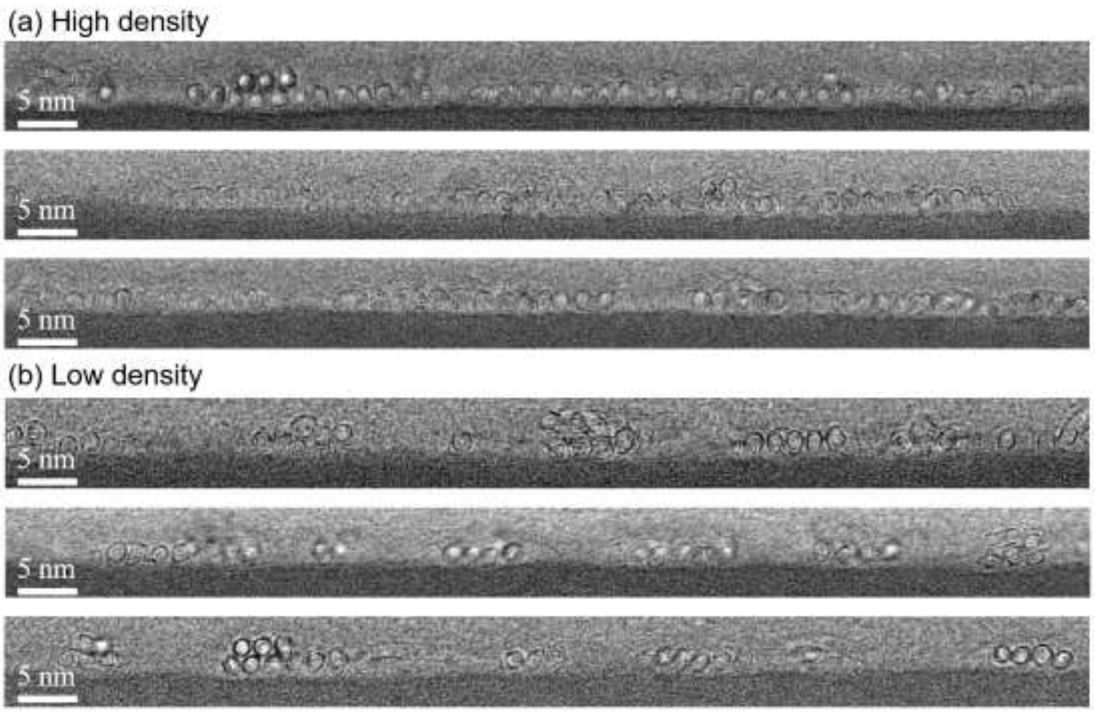

Figure S28. Cross-sectional TEM images of the A-CNT films with high density (b) and low density (a).

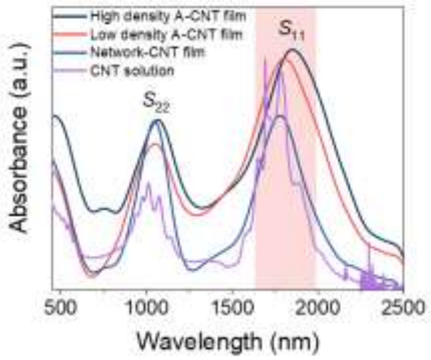

Figure S29. Visible/near-infrared absorption spectrum of the CNT films, including high-density A-CNT films, low-density A-CNT film, network-CNT film, and arc-CNT solution extracted by PCz. The CNT films arranged on the Si/SiO_2 substrate are transferred to a quartz substrate based on a poly(methyl methacrylate) (PMMA)-assisted wet transfer method, before the absorption spectrum (measured by Cary 5000). The resonance absorption peaks are located at ~ 1000 nm and 1800 nm, corresponding to the positions of transition characteristic of semiconducting CNT (S_{22} and S_{11}).

Supplementary Texts 5. Energy scale of intertube coupling

The effects of intertube coupling can be classified into two types. One arises from the electrodynamical coupling in which Coulomb interaction in a CNT is modified by the dielectric screening induced by other adjacent CNTs. The second type of intertube coupling is the coherent tunneling coupling of the electronic states in adjacent CNTs

from the overlap of their wave functions. The energy scale of the electrodynamical coupling is comparable to or larger than thermal fluctuation at room temperature, leading to the redshifts in optical experiments of CNT bundles, including Rayleigh scattering spectroscopy [11], resonance Raman [12,13], and photoluminescence [14]. The energy scale of the coherent tunneling coupling is usually smaller than thermal fluctuation at room temperature, and is related to the microscopic details of the CNT bundles, leading to changes in the electronic structures [15,16]. The tunneling matrix element calculated from the model of Maarouf *et al.* [16] is shown in Fig. S30a, which decreases rapidly with increasing intertube spacing. At an intertube spacing of 3.4 Å, the energy scale of the tunneling coherent coupling is about 7.5 meV, close to that estimated from our transport experiments (< 10 meV). Therefore, the intertube coupling effects on the band structure of A-CNT films driven by the coherent tunneling coupling vanish near 100 K (Fig. 1e) and the pronounced phenomenon is hardly observed at room temperature. Based on the diameter and intertube spacing, the coupling strength of the ideal array at different densities can be estimated, as shown in Fig. S30b. Consequently, the effect of intertube coupling disappears below a density of 500 CNTs/μm at measured temperature 2 K (0.17 meV) with a diameter of 1.5 nm. The numerical analysis above is consistent with the DFT calculation in Fig. 3, where the subband redistribution disappears at an intertube spacing of 5.4 Å, equivalent to a density of 490 CNTs/μm. It is understandable that the intertube coupling effects can be observed in the devices based on low-density A-CNTs, due to the non-ideal alignment [17] and high-κ dielectric environment.

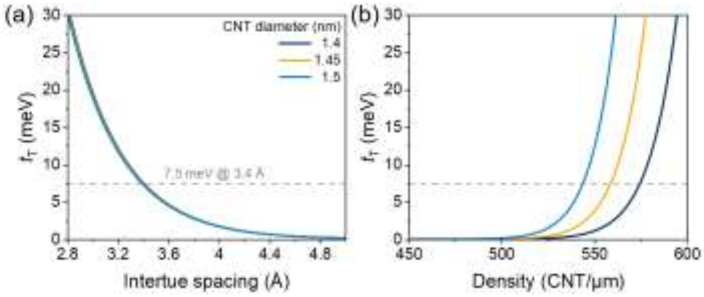

Figure S30. Calculated tunneling matrix element as a function of intertube spacing at various CNT diameters **(a)** and density of A-CNT films **(b)**.

Figure S31a summarizes the positions of S_{11} of all measured absorption spectra. The peak fitting of Viogt line shape is used to extract S_{11} in CNT solution, as shown in Figure S31b. Compared to the S_{11} of CNT solution, the redshifts of about 15 nm (~7 meV) and 40 nm (~16 meV) in S_{11} for network-CNT and A-CNT films arise here, respectively, while the similar S_{11} of high- and low-density A-CNT films. According to the discussion above, the observed redshifts may originate from the variations of the dielectric environment induced by electrodynamical coupling. However, the reductions of band gap may also partly contribute to the redshifts due to the residual effects of coherent tunneling coupling, although the observed redshifts are smaller than thermal fluctuation at room temperature. A clear distinction between the contributions of two

coupling mechanisms requires further experiments, such as optical experiments with variable temperatures.

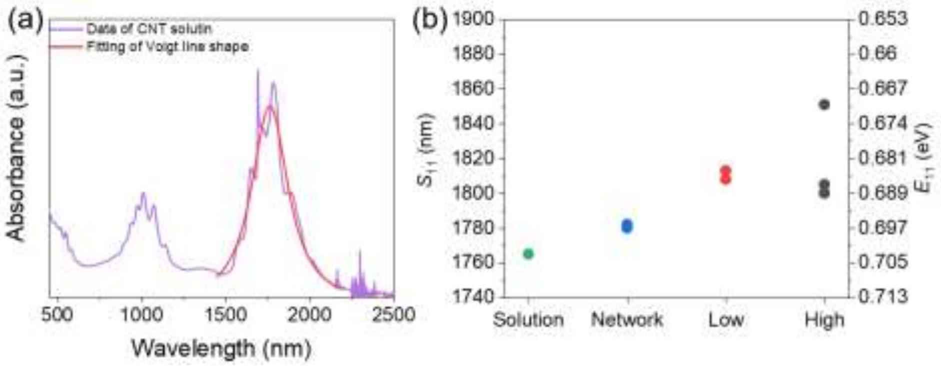

Figure S31. (a) S_{11} extracted from the absorption spectrum of the CNT solution. **(b)** S_{11} of all measured absorption spectra, demonstrating the redshifts.

Next, we discuss the results between DFT calculations and experiments. First, the significant reductions of the band gap come from the DFT-calculated configuration of individual-(19,0) CNT in 2D array. Since the strength of coherent tunneling coupling is related to the microscopic details of the CNT bundles, the variation of the band gap in multichiral CNT systems here can not simply correspond to the single case. The reductions of band gap in all configurations calculated in DFT are extracted by subtracting the band gap of isolated CNT or direct superposition of the band between different CNTs without intertube coupling, as shown in Fig. S32. Cumulative distribution function of the band gap reductions in Fig. S32b demonstrates the median value of 16.6 meV and 60% configurations are smaller than thermal fluctuation at room temperature, while only several configurations possess significantly larger reductions.

Notably, the coherent tunneling between adjacent CNTs is present in all the configurations, as DFT calculations are performed at zero temperature with ideal arrangement. The energy scale can be further reduced by considering the inhomogeneities, orientational disorder, packing efficiency, and more chiralities of the realistic systems [12]. Moreover, the key factor of subband redistribution induced by the intertube coupling is coherence between the adjacent CNTs. No pronounced effects on the band structure can arise from incoherence of intertube coupling, even if the changes in band gap are greater than the thermal fluctuation.

Consequently, no significant reduction of the band gap can be observed at room temperature, since consideration of both thermal fluctuation and realistic CNT systems makes it difficult to preserve the coherence of the intertube coupling, and only several ideal configurations in DFT calculations favor large reductions of the band gap at zero temperature. The redshifts in the absorption spectra may be attributed to residual effects of the band gap reductions, or arise from incoherent electrodynamical coupling between the CNTs.

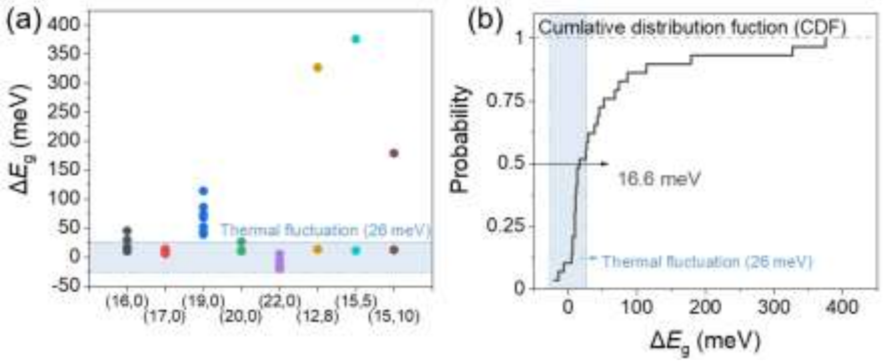

Figure S32. Statistical results **(a)** and cumulative distribution function **(b)** of band gap reductions of various configurations used in DFT calculations. Blue region demonstrates the energy range of thermal fluctuation at room temperature.

References

1. Jorio A, Saito R, Hafner JH *et al.* Structural (n,m) determination of isolated single-wall carbon nanotubes by resonant raman scattering. *Phys Rev Lett* 2001; **86**: 1118-21.
2. Pfohl M, Tune DD, Graf A *et al.* Fitting single-walled carbon nanotube optical spectra. *ACS Omega* 2017; **2**: 1163-71.
3. Kouwenhoven LP, Austing DG, Tarucha S. Few-electron quantum dots. *Rep Prog Phys* 2001; **64**: 701-36.
4. Liu YF, Ding SJ, Li WL *et al.* Interface states in gate stack of carbon nanotube array transistors. *ACS Nano* 2024; **18**: 19086-98.
5. Jarillo-Herrero P, Sapmaz S, Dekker C *et al.* Electron-hole symmetry in a semiconducting carbon nanotube quantum dot. *Nature* 2004; **429**: 389-92.
6. Jespersen TS, Grove-Rasmussen K, Paaske J *et al.* Gate-dependent spin-orbit coupling in multielectron carbon nanotubes. *Nat Phys* 2011; **7**: 348-53.
7. Kresse G, Furthmuller J. Efficient iterative schemes for ab initio total-energy calculations using a plane-wave basis set. *Phys Rev B* 1996; **54**: 11169-86.
8. Blochl PE. Projector augmented-wave method. *Phys Rev B* 1994; **50**: 17953-79.
9. Perdew JP, Burke K, Ernzerhof M. Generalized gradient approximation made simple. *Phys Rev Lett* 1997; **78**: 1396.
10. Grimme S, Ehrlich S, Goerigk L. Effect of the damping function in dispersion corrected density functional theory. *J Comput Chem* 2011; **32**: 1456-65.
11. Wang F, Sfeir MY, Huang LM *et al.* Interactions between individual carbon nanotubes studied by rayleigh scattering spectroscopy. *Phys Rev Lett* 2006; **96**: 167401.
12. O'Connell MJ, Sivaram S, Doorn SK. Near-infrared resonance raman excitation profile studies of single-walled carbon nanotube intertube interactions: A direct comparison of bundled and individually dispersed hipco nanotubes. *Phys Rev B* 2004; **69**: 235415.
13. Fantini C, Jorio A, Souza M *et al.* Optical transition energies for carbon nanotubes from resonant raman spectroscopy: Environment and temperature effects. *Phys Rev Lett* 2004; **93**: 147406.
14. Crochet JJ, Sau JD, Duque JG *et al.* Electrodynamic and excitonic intertube interactions in semiconducting carbon nanotube aggregates. *ACS Nano* 2011; **5**: 2611-8.
15. Reich S, Thomsen C, Ordejon P. Electronic band structure of isolated and bundled carbon nanotubes. *Phys Rev B* 2002; **65**: 155411.

16. Maarouf AA, Kane CL, Mele EJ. Electronic structure of carbon nanotube ropes. *Phys Rev B* 2000; **61**: 11156-65.
17. Wang B, Lu H, Ding S *et al.* Nonideality in arrayed carbon nanotube field effect transistors revealed by high-resolution transmission electron microscopy. *ACS Nano* 2024; **18**: 22474.
